# Serum FT3/FT4 ratio may indicate the degree of T3 compensation in congenital hypothyroidism

**DOI:** 10.1371/journal.pone.0361192

**Published:** 2026-11-11

**Authors:** Takeru Yamauchi, Ami Keidai, Yutaro Tomobe, Seiichi Tomotaki, Kougoro Iwanaga, Junko Takita, Masahiko Kawai

**Affiliations:** Department of Pediatrics, Graduate School of Medicine, Kyoto University, Kyoto, Japan; Consiglio Nazionale delle Ricerche, ITALY

## Abstract

**Background:**

Clinical guidelines for hypothyroidism recommend measuring serum thyroid-stimulating hormone (TSH) and free thyroxine (FT4) levels as diagnostic and treatment efficacy indicators. However, they do not recommend measuring serum free triiodothyronine (FT3) levels because serum FT3 levels often remain within the normal range. However, serum TSH and FT4 levels alone make diagnosis and treatment decision-making difficult in conditions such as central hypothyroidism, suggesting the need for an additional indicator distinct from serum TSH levels. We investigated whether the serum FT3/FT4 ratio could be an indicator of congenital hypothyroidism (CH) severity, instead of serum TSH level.

**Methods:**

Thirty-seven newborns were diagnosed with CH (severe group (n = 13): serum TSH ≥ 30 mIU/L; moderate group (n = 24): serum TSH ≥ 5 to < 30 mIU/L). All infants in the severe group received thyroid hormone (TH) replacement therapy vs 13 infants in the moderate group (moderate group with CH). Serum TSH, FT4, FT3, and FT3/FT4 ratios were compared between each group and the two control groups of different ages at diagnosis and 2 months after treatment.

**Results:**

The serum FT3/FT4 ratio was elevated before treatment compared with the control group, reflecting CH severity. Two months after TH replacement, the serum FT3/FT4 ratio decreased significantly: severe group: at diagnosis, 0.48 vs 2 months, 0.27, *p* =<0.0001; moderate group with TH: 0.38 vs 2 months, 0.26, *p* < 0.0001. No change was observed in infants without TH replacement.

**Discussion:**

A high serum FT3/FT4 ratio in CH could reflect the degree of systemic T3 compensation and is an indicator of CH severity. This ratio may also be applicable in conditions in which serum TSH levels are not useful for diagnosis or when assessing treatment outcomes.

**Conclusion:**

A high serum FT3/FT4 ratio may be an indicator of hypothyroidism severity.

## Introduction

Serum thyroid-stimulating hormone (TSH) and free thyroxine (FT4) are commonly used to diagnose primary hypothyroidism in both adults and children, including the neonatal period [1,2]. In contrast, serum TSH is not effective for diagnosing central hypothyroidism, whereas serum FT4 is regarded as the most reliable marker [3]. The frequency of central hypothyroidism in adults is estimated to be 1/1000 of that in primary hypothyroidism patients [4]. In contrast, central congenital hypothyroidism has an estimated prevalence of 1 in 13,000–30,000 individuals [5], accounting for approximately 1/10 of congenital hypothyroidism cases, making it an underrecognized rather than truly rare condition. However, low serum FT4 levels alone cannot be used to diagnose mild central hypothyroidism [6]. Furthermore, it has long been noted that some patients with hypothyroidism show no improvement in subjective symptoms even when serum TSH levels improve with treatment [7], with this proportion estimated at 15% [8,9]. In addition, even levothyroxine doses sufficient to normalize serum TSH levels do not fully normalize biochemical parameters, such as serum low-density lipoprotein cholesterol levels [10]. Although conventional serum TSH and FT4 measurements are sufficient for the diagnosis and management of most cases of primary hypothyroidism and severe central hypothyroidism, some cases cannot be fully evaluated using these markers alone. Therefore, additional markers that complement serum TSH may help support a more comprehensive evaluation of hypothyroidism diagnosis and treatment response.

In contrast, serum free triiodothyronine (FT3) measurement is not recommended in guidelines [1,2] because serum FT3 levels often remain within the normal range even in patients with hypothyroidism. However, these guidelines do not clearly explain why serum FT3 remains within the reference range. Recent studies suggest that when serum FT4 production per unit of TSH decreases, the hypothalamic-pituitary-thyroid axis prioritizes maintaining serum FT3 levels by increasing the conversion efficiency from FT4 to FT3 over maintaining the set point via TSH and FT4 [11]. Furthermore, in hypothyroidism, the activity of type II deiodinase (D2), which converts T4 to its physiologically active form T3 in peripheral tissues, increases [12]. Therefore, a serum FT3 level within the normal range despite a low serum FT4 level indicates that the thyroid system is compensating by increasing systemic T3 or FT3 production. A relative elevation in serum FT3 concentration compared with serum FT4 concentration may therefore reflect compensatory changes in thyroid hormone regulation that are not fully captured by the absolute serum FT3 or FT4 concentration alone. In this context, evaluating the relationship between these two hormones may provide additional information. Accordingly, an elevated serum FT3/FT4 ratio can be considered an indicator of systemic compensatory tendencies reflecting hypothyroidism severity.

If the above hypothesis is correct, it is necessary to consider whether the serum FT3/FT4 ratio, as with serum TSH levels, could serve as a diagnostic and therapeutic indicator for hypothyroidism. Some cases of hypothyroidism are central hypothyroidism, and TSH cannot be used for diagnosis, requiring alternative diagnostic markers. Therefore, we focused on congenital hypothyroidism (CH), in which thyroid hormone (TH) deficiency is evident and requires urgent replacement therapy. Accordingly, we investigated the similarities and differences in serum FT3/FT4 ratios and TSH levels.

The aim of the current study was to evaluate the usefulness of the FT3/FT4 ratio in assessing hypothyroidism severity. Accordingly, we examined the serum FT3/FT4 ratio prior to treatment in patients with CH. To our knowledge, while previous studies have investigated the serum FT3/FT4 ratio after CH treatment [13,14], none have examined the serum FT3/FT4 ratio before CH treatment. Individuals with CH are considered an optimal group for confirming an elevated FT3/FT4 ratio in hypothyroidism for the following reasons: First, CH has a clearly defined onset period for hypothyroidism, and neonatal screening methods are well-established, resulting in a nearly uniform interval from onset to diagnosis and initiation of treatment. Second, in severe cases, delayed treatment leads to marked psychomotor developmental delay, clearly demonstrating thyroid hormone deficiency [15].

## Methods

### Patients

We retrospectively examined data for 37 term newborns who were suspected of having CH by newborn screening, visited Kyoto University Hospital between April 2005 and December 2021, and were diagnosed as having primary CH (TSH ≥ 5 mIU/L) after close examination. The patients were stratified into three groups using the following classification criteria: The severe CH group (n = 13) was defined as serum TSH ≥ 30.0 mIU/L, and levothyroxine (LT4) treatment was initiated immediately. The other 24 patients (moderate CH group, defined as TSH ≥ 5.0 to < 30 mIU/L) were divided into two groups: a moderate CH with TH group (n = 13) that began LT4 therapy, and a moderate CH with observation group (n = 11) that did not receive LT4 and were followed up. The classification of the severe and moderate CH groups was in accordance with the Guidelines for Newborn Screening of Congenital Hypothyroidism 2021 Revision published by the Japan Pediatric Endocrine Society [16]. Serum TSH, FT4, FT3, and FT3/FT4 ratios were compared between each group and the control group at diagnosis and 2 months after treatment (or 2 months after diagnosis for the moderate CH with observation group). For comparison, the control groups were defined as follows: Control group 1 (n = 20) comprised full-term euthyroid infants aged 7–30 days, with serum TSH concentrations < 5.0 and > 0.4 mIU/L and serum FT4 concentrations ≥ 10 pmol/L. Control group 2 (n = 11) comprised healthy full-term infants aged 2 months, with serum TSH concentrations < 5.0 and > 0.4 mIU/L and serum FT4 concentrations ≥ 10 pmol/L. None of the infants in either control group had thyroid disease or systemic conditions, such as congenital heart disease, central nervous system (CNS) disease, chromosomal abnormalities, or maternal thyroid disease. Control data were obtained from samples collected for the evaluation of prolonged jaundice, weight gain assessments, or infantile hemangiomas. The selection criteria for each control group are described in S1 and S2 Figs. The clinical characteristics of the groups are shown in the Table 1.

**Table 1:** Clinical characteristics of the patients in each group (median, IQR).

	Severe CH (n = 13)	Moderate CH with TH (n = 13)	Moderate CH with observation (n = 11)	Control group 1 (n = 20)	Control group 2 (n = 11)	Kruskal–Wallis *p*-value
Gestational age	40w0d(38w3d–40w5d)	40w0d(38w4d–40w2d)	39w5d(38w1d–40w3d)	38w6d(37w4d–39w4d)	39w1d(38w1d–41w0d)	0.2381
Birth weight (g)	3087(2860–3461)	3070(2722–3154)	2956(2608–3190)	3087(2936–3419)	2800(2550–3486)	0.5660
Female patients, n (%)	8 (62)	8 (62)	6 (55)	8 (40)	7 (64)	0.6296
Day of diagnosis*	15 (11–18.5)	24 (20–28)	21 (17–23)	12 (9–15)		<0.0001
Days from diagnosis to next outpatient visit **	10 (7–14)	14 (14–15)	14 (14–21)			0.0076
Age on the day of examination at 2 months (days)	69 (63.5–81)	82 (66–89)	72 (66–84)		75 (66–84)	0.4683

*: Multiple comparisons revealed significant differences between the severe CH and moderate CH with TH groups (*p* = 0.0051); moderate CH with TH group and control group 1 (*p* = 0.0002); and moderate CH with observation group and control group 1 (*p* = 0.0006).

**: Multiple comparisons revealed significant differences between the severe CH and moderate CH with TH groups (*p* = 0.0055).

IQR, interquartile range; CH, congenital hypothyroidism; w, weeks; d, days

This study was performed in accordance with the ethical principles of the Declaration of Helsinki, as revised in 2013, and with the approval of the Kyoto University Graduate School and Faculty of Medicine Ethics Committee (approval number: R3348). Ethical considerations in our research include allowing participants (or their parents or guardians) to opt out if they do not wish to continue participation. To conduct this study, medical records were accessed, and data were collected from December 23, 2022, to July 12, 2024. Data collection and analysis were conducted following anonymization to ensure the exclusion of any personally identifiable information.

### Thyroid function testing

Serum TSH, FT3, and FT4 concentrations and the FT3/FT4 ratios in the three CH groups were evaluated at the time of diagnosis and approximately 2 months after the start of treatment. From April 2005 to December 2021, measurements were obtained using electrochemiluminescence kits: FT3 by Elecsys FT3 III and FT4 by Elecsys FT4 IV (Roche Diagnostics, Mannheim, Germany). Harmonization of serum TSH concentrations was performed using coefficients published by the Japanese Society for Laboratory Medicine [17].

### Statistical analysis

Statistical analyses were performed using JMP version 17 (SAS Institute Inc., Cary, NC, USA). Statistical analyses were performed using the Kruskal–Wallis test, with the Wilcoxon rank-sum test with Bonferroni correction for multiple comparisons. Changes in serum FT3/FT4 ratios were assessed for each patient at the time of diagnosis and 2 months later using the Wilcoxon signed-rank test. The results are presented as median and interquartile range for each group. Statistical significance was set at 0.05, and the significance level for multiple comparisons with Bonferroni correction was set at 0.0083.

## Results

### Patients’ characteristics

The patient enrollment process is shown in Fig 1, and the characteristics of the three CH and two control groups are shown in the Table 1. Each group comprised only full-term neonates. The day of diagnosis was significantly later in the moderate CH with TH group and moderate CH with observation group than in the severe CH group, and the LT4 dose (μg/kg) was significantly higher in the severe CH group (LT4 dose: severe CH, 9.69 [9.10–10.00]; moderate CH with TH, 5.00 [4.62–6.53]).

**Fig 1:**
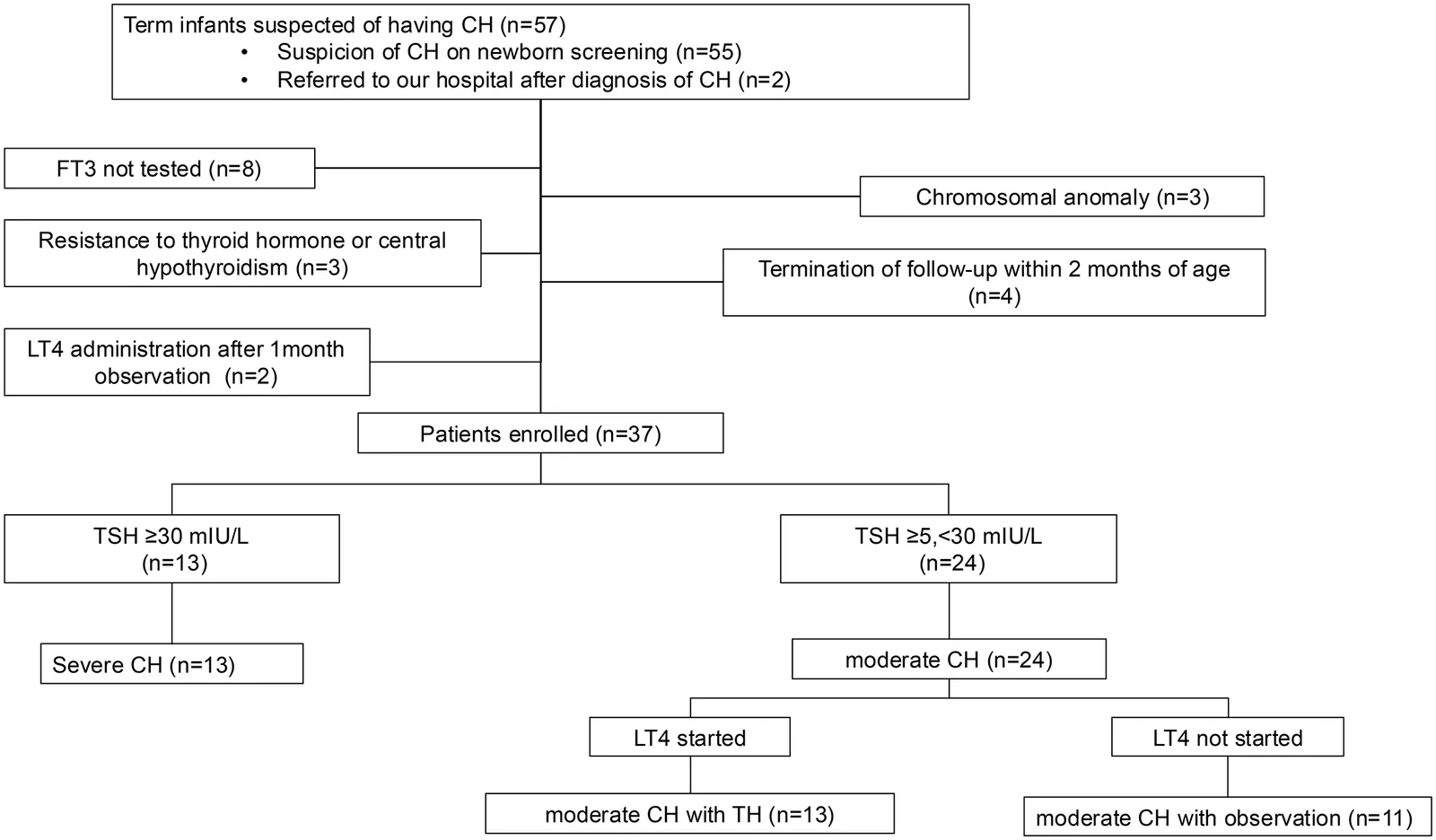
Flowchart of the enrollment process. Of the 57 patients with suspected CH, 20 were excluded on the basis of the following criteria: FT3 not tested, presence of chromosomal abnormalities, TH resistance and central hypothyroidism, and follow-up < 2 months. Therefore, 37 patients were enrolled in the present study. CH, congenital hypothyroidism; FT3, free triiodothyronine; TH, thyroid hormone.

### Thyroid function parameters at diagnosis

We compared serum TSH, FT4, and FT3 concentrations, and the FT3/FT4 ratios at diagnosis between the severe CH group, moderate CH with TH group, moderate CH with observation group, and control group 1. The Kruskal–Wallis test showed significant differences among the groups in serum TSH, FT4, and FT3 concentrations and in the FT3/FT4 ratio (*p* < .05) (Fig 2A). Serum FT4 concentrations were significantly different in each group except for the moderate CH with TH group and the moderate CH with observation group (Fig 2B). The Kruskal–Wallis test showed a significant difference in serum FT3 concentrations (Fig 2C), but multiple comparisons showed no significant differences between the groups. The serum FT3/FT4 ratio differed significantly between the groups except for the moderate CH with TH group and the moderate CH with observation group (severe CH: 0.48 (0.42–0.65), moderate CH with TH: 0.38 (0.34–0.42), moderate CH with observation: 0.33 (0.30–0.37), control group 1: 0.24 (0.20–0.27); *p* < 0.0001) (Fig 2D; S1 and S2 Tables). The correlation between serum TSH concentration and the FT3/FT4 ratio is shown in S3 Fig (*r* = 0.56, *p* < 0.0001).

**Fig 2:**
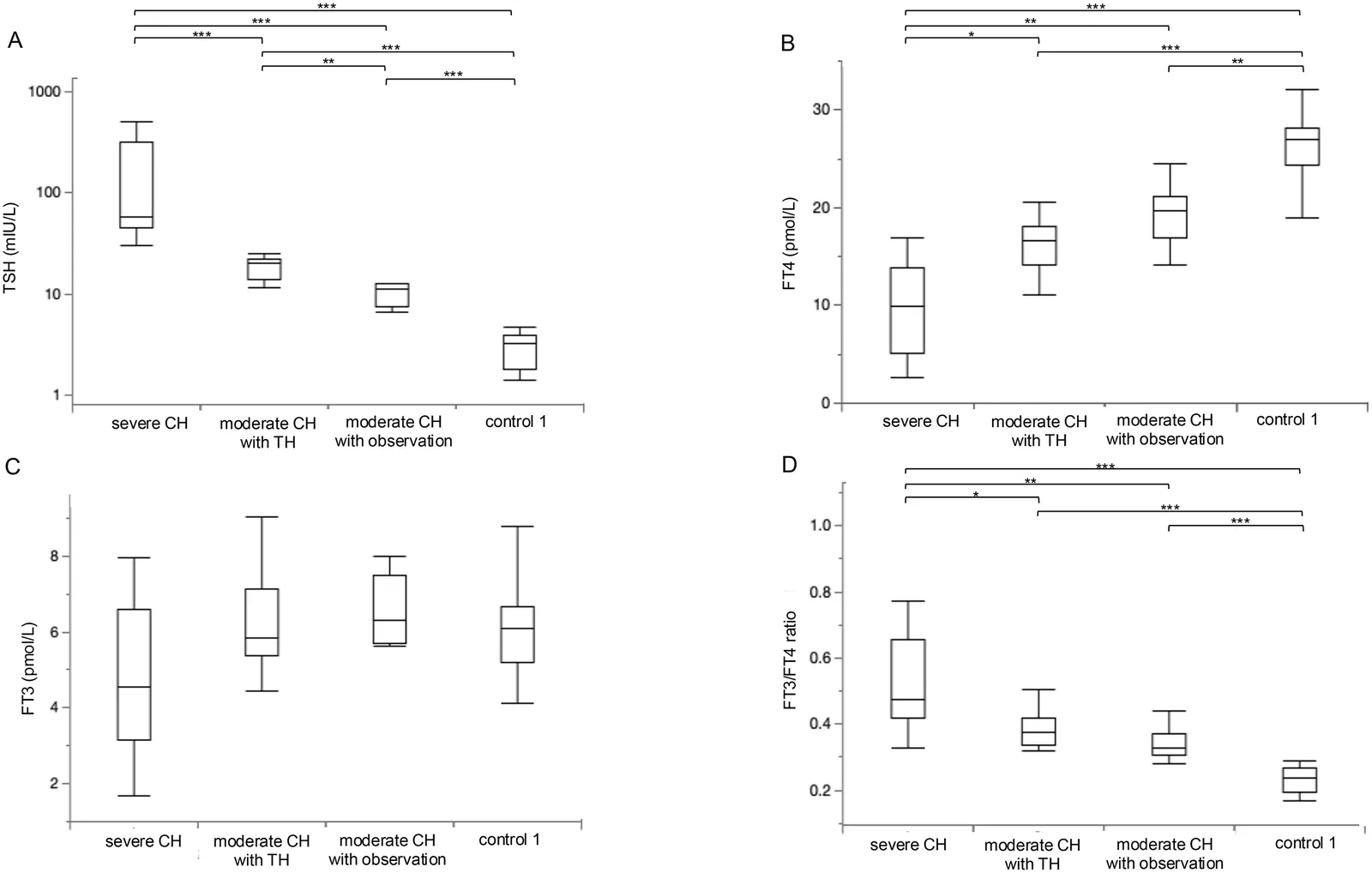
Serum TSH, FT4, and FT3 concentrations, and FT3/FT4 ratios between the groups at diagnosis. A) TSH, B) FT4, C) FT3, and D) FT3/FT4 ratio. In accordance with the classification criteria for CH, serum TSH and FT4 concentrations differed depending on the severity of thyroid dysfunction (A, B). In contrast, the serum FT3 concentration showed no significant differences between the groups (C). The FT3/FT4 ratio increased with hypothyroidism severity (D). The top, bottom, and middle lines in the boxes correspond to the 75th, 25th, and 50th percentile (median), respectively. TSH, thyroid-stimulating hormone; FT4, free thyroxine; FT3, free triiodothyronine; FT3/FT4 ratio, ratio of free T3 to free T4; CH, congenital hypothyroidism; ***, *p* < 0.0001; **, *p* < .001; *, *p* < .01; NS, not significant.

### Thyroid function parameters after 2 months of treatment and comparisons of the serum FT3/FT4 ratios before and after treatment

We compared serum TSH, FT4, and FT3 concentrations, and the FT3/FT4 ratios 2 months post-treatment between the severe CH group, moderate CH with TH group, moderate CH with observation group, and control group 2. The Kruskal–Wallis test showed significant differences between the groups for serum TSH, FT4 and FT3 concentrations, and the FT3/FT4 ratio (*p* < 0.05). Multiple comparisons were performed between the three groups using the Bonferroni method. The moderate CH with observation group had significantly higher serum TSH concentrations than those in the other groups (Fig 3A). There was no significant difference in serum TSH concentrations between the other groups. The serum FT4 concentrations in the severe CH and moderate CH with TH groups were significantly higher than those in the moderate CH with observation group (Fig 3B). No significant difference was observed between the moderate CH with observation group and control group 2. Furthermore, no significant differences were found between the severe CH group and control group 2. Serum FT3 concentrations differed significantly between the moderate CH with TH group and control group 2. There were no statistically significant differences in serum FT3 concentrations between any of the other groups (Fig 3C). The serum FT3/FT4 ratio was significantly lower in both the severe and moderate CH with TH groups than those in the moderate CH with observation group and control group 2 (Fig 3D). However, no statistical difference was detected between the severe and moderate CH with TH groups or between the moderate CH with observation group and control group 2 (S3 and S4 Tables).

**Fig 3:**
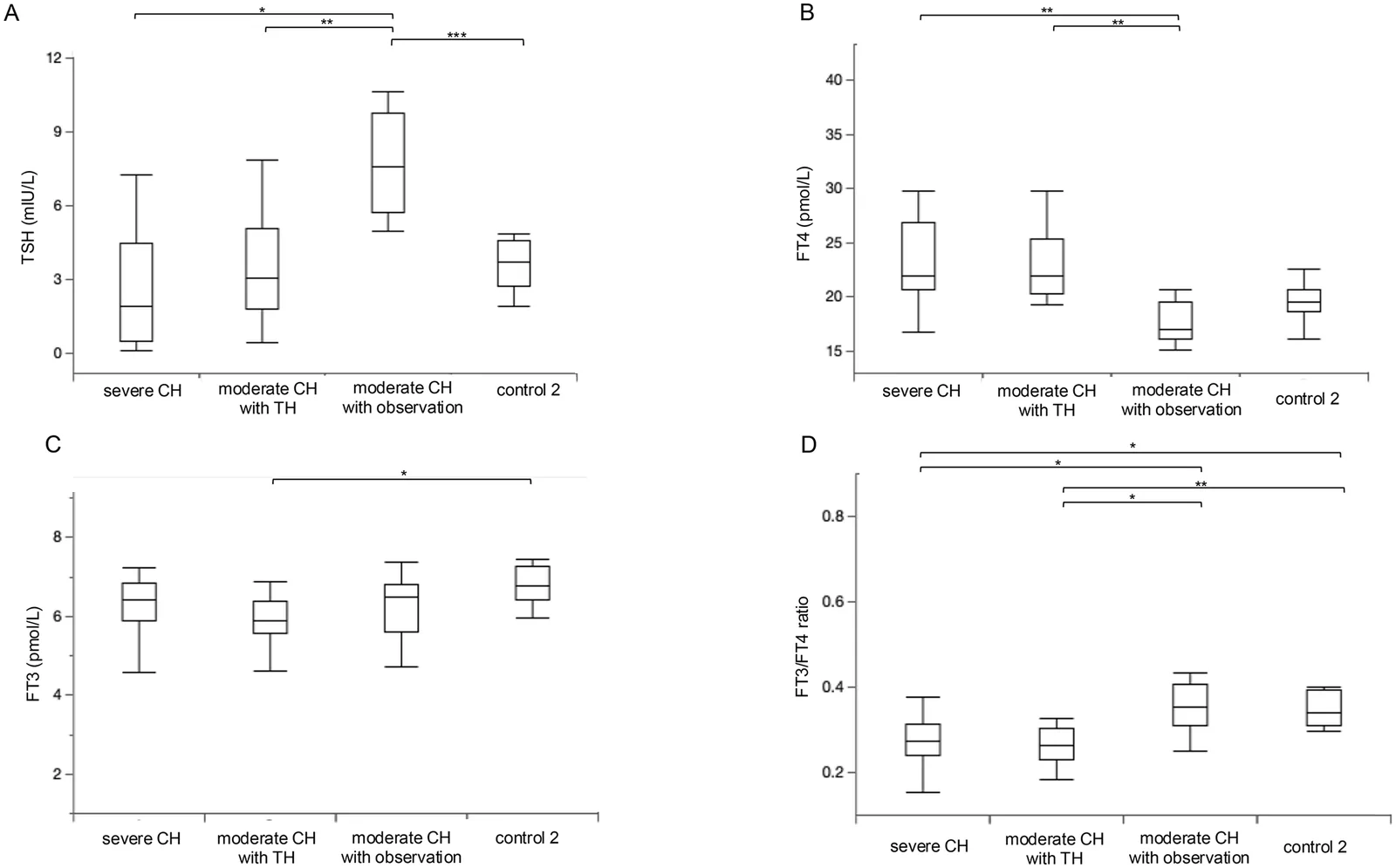
Serum TSH, FT4, and FT3 concentrations, and FT3/FT4 ratios between the groups at 2 months. A) TSH, B) FT4, C) FT3, and D) FT3/FT4 ratios. In both the severe and moderate CH with TH groups, LT4 administration reduced serum TSH concentrations to the same level as those of control group 2 (A), and serum FT4 concentrations were significantly higher than those of control group 2 (B). In the moderate CH with observation group, TSH concentrations remained elevated, and FT4 concentrations were similar to those in control group 2 (A, B). A statistically significant difference in serum FT3 concentrations was observed between the moderate CH with TH group and control group 2. There were no statistically significant differences in serum FT3 concentrations between any of the groups (C). Reflecting these improvements, the FT3/FT4 ratios of the severe and moderate CH groups were both significantly lower than those of control group 2 (D). The FT3/FT4 ratios in the CH observation group were similar to those of control group 2. The top, bottom, and middle lines in the boxes correspond to the 75th, 25th, and 50th percentiles (median), respectively. TSH, thyroid stimulating hormone; FT4, free thyroxine; FT3, free triiodothyronine; FT3/FT4 ratio, ratio of free T3 to free T4; LT4, levothyroxine; TH, thyroid hormone; CH, congenital hypothyroidism; ***, *p* < 0.0001; **, *p* < 0.001; *, *p* < 0.01; NS, not significant.

### FT3/FT4 ratio changes in the groups treated with TH

The serum FT3/FT4 ratio at diagnosis and 2 months after the start of treatment were compared for the severe CH and moderate CH with TH groups. In both the severe and moderate CH with TH groups, the serum FT3/FT4 ratio decreased significantly after treatment: severe CH: at diagnosis, 0.48 (0.42–0.65) vs 2 months, 0.27 (0.24–0.31), *p* < 0.0001 and moderate CH with TH: at diagnosis, 0.38 (0.34–0.42) vs 2 months, 0.26 (0.23–0.31), *p* < 0.0001) (Fig 4A–C).

**Fig 4:**
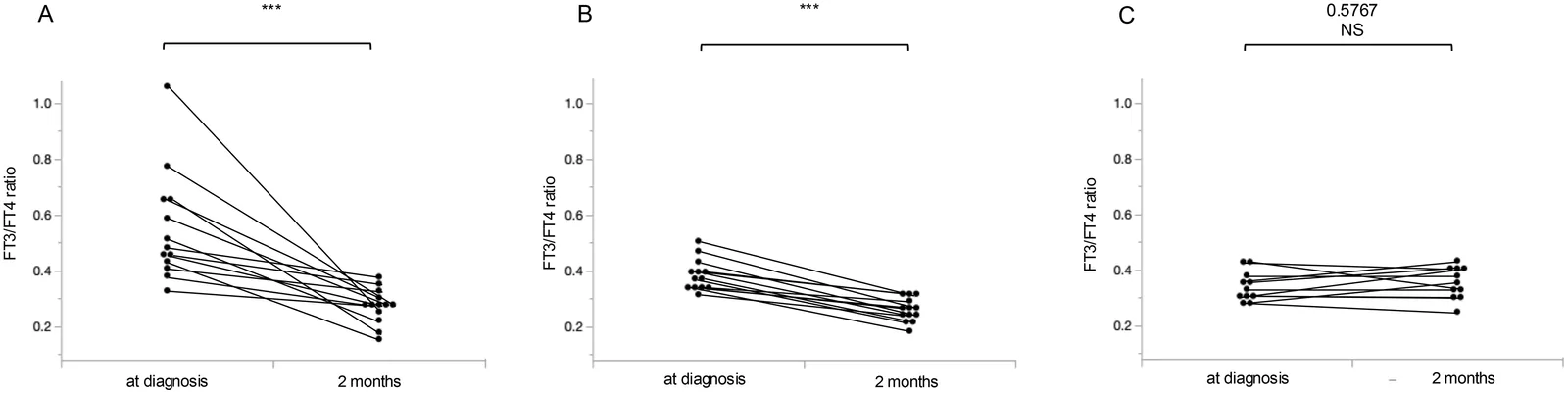
Changes in the serum FT3/FT4 ratio in the severe (A), moderate CH with TH (B), and moderate CH with observation (C) groups at diagnosis and 2 months after LT4 administration or follow-up. Both LT4-treated groups (severe and moderate CH with TH groups) showed a decrease in serum FT3/FT4 ratio after the start of treatment, while the moderate CH with observation group showed no significant decrease in the serum FT3/FT4 ratio at the 2-month follow-up. FT3, free triiodothyronine; FT4, free thyroxine; LT4, thyroxine; CH, congenital hypothyroidism; TH, thyroid hormone; FT3/FT4 ratio, ratio of free T3 to free T4; ***, *p* < 0.0001; NS, not significant.

### Receiver operating characteristic curve for the serum FT3/FT4 ratio at diagnosis

To explore the potential cutoff value of the pretreatment serum FT3/FT4 ratio for distinguishing the CH groups (severe CH, moderate CH with TH, and moderate CH with observation) from Control group 1, a receiver operating characteristic (ROC) curve was constructed (S4 Fig). This analysis suggested a tentative cutoff value of 0.30, with an area under the curve (AUC) of 0.99, sensitivity of 94.59%, and specificity of 100% (S4 Fig).

### Comparison of the FT3/FT4 ratio and TSH for the moderate CH with observation group and the control groups

The serum FT3/FT4 ratios in the moderate CH with observation group were not significantly different between diagnosis and at 2 months (at diagnosis, 0.33 (0.30–0.37) vs 2 months, 0.35 (0.31–0.41), *p* = .52; Fig 4C). However, as mentioned, in the moderate CH with observation group, the serum FT3/FT4 ratio at diagnosis was significantly higher than that in control group 1, whereas the serum FT3/FT4 ratio at 2 months was not significantly different from that of control group 2 (Figs 2D and 3D). Furthermore, serum TSH concentrations at 2 months in the moderate CH with observation group were significantly lower than those at diagnosis (at diagnosis, 10.83 (7.39–12.21) vs 2 months, 7.57 (5.69–9.75), *p* = 0.0235; S5 Fig).

## Discussion

In the current study, a high serum FT3/FT4 ratio was associated with CH severity, as assessed by serum TSH concentration. Serum FT4 concentrations were significantly lower in the severe CH group than in the other groups. Whereas, serum FT3 concentrations did not differ significantly among the groups. The findings of this study suggest that in settings where serum TSH is unreliable, an elevated FT3/FT4 ratio could support a diagnosis of undertreated or untreated hypothyroidism. Specifically, these findings imply that under TH-deficient conditions, increased deiodinase activity and enhanced T4-to-T3 conversion may help to compensate for T3 deficiency. T3 metabolism depends on the type 1 and 2 deiodinases (D1 and D2), which convert T4 into the active hormone, T3 [18]. In individuals with normal thyroid function, approximately 80% of T3 production occurs through extrathyroidal conversion of T4 [19]. Whereas the remaining 20% is generated in the thyroid gland. D2 is the major source of extrathyroidal T3 in humans and is thought to play an important role in hypothalamic–pituitary regulation [20]. T3 generation by D2 occurs in the perinuclear region and contributes to TH receptor occupancy. Whereas, D1 localizes to the plasma membrane and is more likely to contribute to plasma T3 production [20]. In hypothyroidism, although thyroid-derived circulating T3 concentrations decrease, peripheral tissue-derived T3 production is thought to increase, thereby delaying decrease in serum and tissue T3 concentrations [21]. Elevated TSH in hypothyroidism may also directly stimulates T3 production within the thyroid gland [11,22]. Therefore, compensatory T3 production is likely to occur in hypothyroidism, and serum FT3 concentration may be maintained if peripheral tissues compensate for reduced thyroidal T3 production.

In the present patients with primary CH, the serum FT3/FT4 ratio tended to increase with hypothyroidism severity. This suggests that greater CH severity may be associated with greater compensatory activation of T3 production. Therefore, in untreated hypothyroidism, the serum FT3/FT4 ratio may serve as an indicator of systemic T3 compensation. In contrast, the serum FT3/FT4 ratio has been reported to be lower in patients with hypothyroidism receiving LT4 than that in healthy individuals [23–25]. Some researchers have suggested that this reflects inadequate peripheral deiodination to compensate for the lack of thyroidal T3 secretion [24], and LT4 monotherapy has therefore been considered insufficient by some authors [23]. However, given that both D1- and D2-mediated peripheral T3 production and thyroid-derived T3 production are also both increased in hypothyroidism, the decrease in the serum FT3/FT4 ratio after LT4 administration may not be attributable solely to T3 deficiency.

A report by Oto et al. may support this interpretation. They compared the FT3/FT4 ratio in patients with congenital hypothyroidism due to thyroid aplasia or hypoplasia who were treated with LT4, for whom serum TSH concentration appeared to be used as the indicator for LT4 adjustment, with that in patients with central hypothyroidism who were also treated with LT4, for whom serum FT4 concentration appeared to be used as the indicator for LT4 adjustment [13]. Patients with central hypothyroidism had a higher FT3/FT4 ratio during treatment. Accordingly, a decrease in the serum FT3/FT4 ratio during LT4 administration, when TSH normalization is used as the treatment indicator might suggest LT4 overtreatment.

Taken together, these findings could suggest that an increased serum FT3/FT4 ratio in patients with primary hypothyroidism, including CH, may reflect systemic TH deficiency beyond the hypothalamic–pituitary axis. Conversely, the decrease in the serum FT3/FT4 ratio after LT4 administration might reflect potentially suppression of T4-to-T3 conversion to avoid systemic TH excess. Thus, a decrease in serum FT3 concentration during LT4 treatment aimed at TSH normalization may not necessarily indicate insufficient treatment; rather, it could potentially indicate that circulating T4 exceeds systemic TH requirements.

The clinical utility of the serum FT3/FT4 ratio should be interpreted cautiously. Because the serum FT3/FT4 ratio correlated with serum TSH severity in this study, its usefulness in primary CH, for which severity can generally be assessed using serum TSH, is likely limited. In particular, the ratio is unlikely to alter clinical management in severe cases with clearly elevated serum TSH levels. In the moderate CH with observation group, serum TSH remained significantly elevated at 2 months, whereas the serum FT3/FT4 ratio did not differ significantly from that in the control group 2. These findings suggest that serum TSH may be a more sensitive marker of primary hypothyroidism. In contrast, the finding that the serum FT3/FT4 ratio increased in proportion to serum TSH elevation in neonatal hypothyroidism suggests that this index may have potential value in settings where serum TSH is less informative, such as central congenital hypothyroidism. Screening based on serum FT4 alone may miss mild-to-moderate central congenital hypothyroidism when the decrease in serum FT4 is slight. Therefore, the serum FT3/FT4 ratio may provide additional information for screening in such cases. However, the present study cannot definitively establish its utility for central congenital hypothyroidism or for mild primary CH, in which the need for treatment remains uncertain. Further studies are required to confirm this hypothesis. In addition, because the sample size of the present study was too small to establish definitive cutoff values, caution is warranted regarding their clinical application. If validated in large-scale datasets, such cutoff values could help inform therapeutic decision-making for mild central congenital hypothyroidism.

This study has several limitations. First, it was a retrospective study performed at a single hospital, which limited the number of patients available for analysis. Second, we did not evaluate CH disease type. Although, the serum FT3/FT4 ratio generally reflected hypothyroidism severity or the degree of systemic T3 compensation, in patients with CH, the ratio may differ according to CH disease type. Thyroglobulin may be useful for inferring disease type but was not measured in all cases. Third, serum reverse T3 concentrations were not measured. Evaluating changes in reverse T3 before and after treatment in patients with CH may provide additional insight into peripheral T3 compensation and the mechanisms underlying the decrease in the serum FT3/FT4 ratio after LT4 administration. Finally, the control groups cannot be definitively classified as healthy newborns. However, the control infants presented with mild jaundice that improved with phototherapy, relatively small hemangiomas, or poor weight gain without other findings, and no abnormalities were detected in standard thyroid function tests such as serum TSH and FT4. Although abnormalities in thyroid hormone metabolism cannot be completely excluded, a marked abnormality in FT4-to-FT3 conversion would be expected to cause thyroid dysfunction, such as elevated serum TSH. Therefore, these infants were considered unlikely to have marked abnormalities in thyroid hormone metabolism and were included as the control group in this study.

## Conclusion

The current study revealed a correlation between CH severity and the serum FT3/FT4 ratio, and that the serum FT3/FT4 ratio decreased after LT4 administration. Therefore, in patients with untreated hypothyroidism, the serum FT3/FT4 ratio may be considered an indicator of the degree of T3 compensation. Furthermore, this finding may be useful in the diagnosis of hypothyroidism, which cannot be diagnosed using TSH alone, such as in central hypothyroidism.

## Supporting information

S1 Table Serum TSH, FT4, and FT4 concentrations, and FT3/FT4 ratios at diagnosis (top section: median, IQR, bottom section: SE). (PDF)

S2 Table p-values for each multiple comparison at diagnosis (Bonferroni method*, top section) and 95% confidence interval (bottom section). (PDF)

S3 Table Serum TSH, FT4, and FT4 concentrations, and FT3/FT4 ratios at 2 months (top section: median, IQR, bottom section: SE). (PDF)

S4 Table p-value for each multiple comparison at 2 months (Bonferroni method*, top section) and 95% confidence interval (bottom section). (PDF)

S1 Fig Flowchart of the selection process of control group 1. Of the 74 term infants aged 7–30 days with TSH, FT4, and FT3 measured on the same day, infants were excluded on the basis of the following criteria: serum TSH > 5.0 mIU/L or < 0.4 mIU/L, birth weight < 2500 g, or the presence of congenital anomalies or neurological disorders, congenital heart disease, gastrointestinal or surgical diseases, endocrine and metabolic disease, and hematologic or immunologic diseases. Therefore, 20 infants were selected as control group 1. a, Infants whose laboratory values were measured multiple times and met the criteria at least once were excluded; b, including infants born to mothers with Grave’s disease; TSH, thyroid stimulating hormone; FT4, free thyroxine; FT3, free triiodothyronine.
(PDF)

S2 Fig Flowchart of the selection process of control group 2. Of the 35 term infants aged 2 months with TSH, FT4, and FT3 measured on the same day, infants were excluded on the basis of the following criteria: serum TSH > 5.0 mIU/L or < 0.4 mIU/L, or the presence of congenital anomalies or neurological disorders, congenital heart disease, gastrointestinal or surgical diseases, endocrine and metabolic disease, and hematologic or immunologic diseases Therefore, 11 infants were selected as control group 2. a, Infants whose laboratory values were measured multiple times and met the criteria at least on ce were excluded; b, including infants born to mothers with Grave’s disease; TSH, thyroid stimulating hormone; FT4, free thyroxine; FT3, free triiodothyronine.
(PDF)

S3 Fig Scatter diagram of serum TSH concentrations and FT3/FT4 ratios. The correlation coefficient was *r* = 0.56 (*p* < 0.0001), indicating a moderate correlation. TSH, thyroid stimulating hormone; FT3, free triiodothyronine; FT4, free thyroxine; FT3/FT4 ratio, ratio of free T3 to free T4.
(PDF)

S4 Fig Receiver operating characteristic (ROC) curve for serum FT3/FT4 ratio to predict CH*. Area Under the Curve is 0.99 *; CH includes severe, moderate with LT4, and moderate with observation.
(PDF)

S5 Fig Changes in TSH concentrations at diagnosis and after 2 months in the moderate CH with observation group. No treatment was administered, but TSH concentrations decreased over 2 months. CH, congenital hypothyroidism; TSH, thyroid stimulating hormone.
(PDF)
