# Supplementary material for: Serum FT3/FT4 ratio may indicate the degree of T3 compensation in congenital hypothyroidism
Source: PLoS One. 2026 Nov 11;21(11):e0361192. doi: 10.1371/journal.pone.0361192 (PMC13754123; doi:10.1371/journal.pone.0361192)
Supplement: Supplementary file 1 [file pone.0361192.s001.pdf]

Supplemental Table 1 Serum TSH, FT4, and FT4 concentrations, and FT3/FT4 ratios at diagnosis (top section: median, IQR, bottom section: SE)

	Severe CH (n=13)	Moderate CH with TH (n=13)	Moderate CH with observation (n=11)	Control group 1 (n=20)	Kruskal–Wallis p-value
TSH (mIU/L)*	56.24 (44.25–312.13)	19.44 (13.66–21.08)	10.83 (7.39–12.21)	3.19 (1.73–3.79)	<0.0001
	65.29	1.26	1.52	0.24	
FT4 (pmol/L)**	9.96 (5.14–13.84)	16.60 (14.16–18.15)	19.69 (16.99–21.11)	26.96 (24.35–28.12)	<0.0001
	1.40	0.76	0.89	0.75	
FT3 (pmol/L)***	4.56 (3.14–6.60)	5.82 (5.38–7.13)	6.31 (5.70–7.48)	6.10 (5.18–6.65)	0.07
	0.57	0.35	0.27	0.24	
FT3/FT4 ratio**	0.48 (0.42–0.65)	0.38 (0.34–0.42)	0.33 (0.30–0.37)	0.24 (0.20–0.27)	<0.0001
	0.06	0.02	0.02	0.01	

*: Significant differences were observed between the groups in multiple comparisons****.

**: Significant differences were observed between the severe and moderate CH with TH groups; severe and moderate CH with observation groups; severe CH group and control group 1; moderate CH with TH group and control group 1; and moderate CH with observation group and control group 1 in multiple comparisons.

***: No significant differences were observed between the groups in multiple comparisons.

****: Please see Supplemental Table 2 for each p-value for multiple comparisons.

TSH, thyroid stimulating hormone; FT4, free thyroxine; FT3, free triiodothyronine; FT3/FT4 ratio, ratio of free T3 to free T4;
IQR, interquartile range; SE, standard error; CH, congenital hypothyroidism
