# Supplementary material for: Serum FT3/FT4 ratio may indicate the degree of T3 compensation in congenital hypothyroidism
Source: PLoS One. 2026 Nov 11;21(11):e0361192. doi: 10.1371/journal.pone.0361192 (PMC13754123; doi:10.1371/journal.pone.0361192)
Supplement: Supplementary file 2 [file pone.0361192.s002.pdf]

Supplemental Table 2 p-values for each multiple comparison at diagnosis (Bonferroni method*, top section) and 95% confidence interval (bottom section)

	Severe CH vs Moderate CH with TH	Severe CH vs Moderate CH with observation	Severe CH vs Control group 1	Moderate CH with TH vs Moderate CH with observation	Moderate CH with TH vs Control group 1	Moderate CH with observation vs Control group 1
TSH	<0.0001	<0.0001	<0.0001	0.0014	<0.0001	<0.0001
	-213.08 – -28.24	-221.69 – -32.99	-226.25 – -47.27	-12.01 – -3.02	-18.58 – -11.53	-9.09 – -5.33
FT4	0.0012	0.0001	<0.0001	0.0161 NS	<0.0001	0.0001
	2.57 – 11.48	5.66 – 14.53	13.26 – 21.36	0.51 – 5.53	7.59 – 12.10	4.25 – 9.40
FT3	0.0577 NS	0.0256 NS	0.0577 NS	0.3848 NS	0.9706 NS	0.0256 NS
	-0.12 – -3.12	0.23 – 3.64	-0.15 – 2.80	-0.38 – 1.37	-1.08 – 0.72	-1.35 – 0.38
FT3/FT4	0.0041	0.0004	<0.0001	.0559 NS	<0.0001	<0.0001
ratio	-0.26 – -0.04	-0.30 – -0.07	-0.39 – -0.20	-0.08 – -0.01	-0.19 – -0.11	-0.15 – -0.07

*: The significance level for multiple comparisons with Bonferroni correction was set at $p<0.0083$.

TSH, thyroid stimulating hormone; FT4, free thyroxine; FT3, free triiodothyronine; FT3/FT4 ratio, ratio of free T3 to free T4; CH, congenital hypothyroidism, NS; not significant
