# Supplementary material for: Serum FT3/FT4 ratio may indicate the degree of T3 compensation in congenital hypothyroidism
Source: PLoS One. 2026 Nov 11;21(11):e0361192. doi: 10.1371/journal.pone.0361192 (PMC13754123; doi:10.1371/journal.pone.0361192)
Supplement: Supplementary file 3 [file pone.0361192.s003.pdf]

Supplemental Table 3 Serum TSH, FT4, and FT4 concentrations, and FT3/FT4 ratios at 2 months. (top section: median, IQR, bottom section: SE)

	Severe CH (n=13)	Moderate CH with TH (n=13)	Moderate CH with observation (n=11)	Control group 2 (n=11)	Kruskal–Wallis p-value
TSH (mIU/L)*	1.90 (0.48–4.46)	3.04 (1.78–5.04)	7.57 (5.69–9.75)	3.70 (2.69–4.59)	<0.0001
	0.93	0.57	0.66	0.32	
FT4 (pmol/L)**	21.88 (20.66–26.83)	21.88 (20.33–25.29)	16.98 (16.09–19.56)	19.56 (18.66–20.72)	<0.0001
	1.74	0.85	0.55	0.51	
FT3 (pmol/L)***	6.42 (5.90–6.86)	5.90 (5.55–6.37)	6.48 (6.42–7.28)	6.78 (6.42–7.28)	0.0131
	0.20	0.16	0.24	0.53	
FT3/FT4 ratio****	0.27 (0.24–0.31)	0.26 (0.23–0.30)	0.35 (0.31–0.41)	0.34 (0.31–0.39)	0.0001
	0.02	0.01	0.02	0.01	

*: Significant differences were observed between the severe CH group and moderate CH with observation group; moderate CH with TH group and moderate CH with observation group; and moderate CH with observation group and control group 2 in multiple comparisons*****.

**: Significant differences were observed between the severe group and moderate CH with observation group and moderate CH with TH group and moderate CH with observation group in multiple comparisons.

***: Significant differences were observed between the moderate CH with TH group and control group 2 in multiple comparisons.

****: Significant differences were observed between the severe and moderate CH with observation groups; severe CH group and control group 2; moderate CH with TH group and moderate CH with observation group; and moderate CH with THgroup and control group 2in multiple comparisons.

*****: Please see Supplemental Table 4 for each p-value for multiple comparisons.

TSH, thyroid stimulating hormone; FT4, free thyroxine; FT3, free triiodothyronine; FT3/FT4 ratio, ratio of free T3 to free T4;
IQR, interquartile range; SE, standard error; CH, congenital hypothyroidism
