# Supplementary material for: Serum FT3/FT4 ratio may indicate the degree of T3 compensation in congenital hypothyroidism
Source: PLoS One. 2026 Nov 11;21(11):e0361192. doi: 10.1371/journal.pone.0361192 (PMC13754123; doi:10.1371/journal.pone.0361192)
Supplement: Supplementary file 4 [file pone.0361192.s004.pdf]

Supplemental Table 4 p-value for each multiple comparison at 2 months (Bonferroni method*, top section) and 95% confidence interval (bottom section)

	Severe CH vs Moderate CH with TH	Severe CH vs Moderate CH with observation	Severe CH vs Control group 2	Moderate CH with TH vs Moderate CH with observation	Moderate CH with TH vs Control group 2	Moderate CH with observation vs Control group 2
TSH	0.2184 NS	0.0021	0.1110 NS	0.0006	0.7280 NS	<0.0001
	-0.84 – 2.69	3.07 – -7.67	-0.73 – 2.92	2.43 – 6.36	-1.24 – 1.68	-6.04 – -2.11
FT4	0.7004 NS	0.0006	0.0174 NS	0.0002	0.0107 NS	0.0193 NS
	-4.25 – 2.96	-8.88 – -2.57	-6.94 – -0.77	-7.72 – -2.96	-5.14 – -0.39	0.39 – 3.73
FT3	0.0724 NS	0.6850 NS	0.1109 NS	0.2583 NS	0.0014	0.0761 NS
	-1.04 – 0.08	-0.80 – 0.57	-0.15 – 0.91	-0.36 – -0.94	0.36 – 1.39	-0.03 – 1.27
FT3/FT4	0.3833 NS	0.0065	0.0038	0.0010 NS	0.0004	0.6458 NS
ratio	-0.05 – 0.03	0.03 – 0.14	0.02 – 0.12	0.05 – 0.14	0.05 – 0.13	-0.06 – 0.04

*: The significance level for multiple comparisons with Bonferroni correction was set at $p<0.0083$.

TSH, thyroid stimulating hormone; FT4, free thyroxine; FT3, free triiodothyronine; FT3/FT4 ratio, ratio of free T3 to free T4; IQR, interquartile range; CH, congenital hypothyroidism, NS; not significan
