# Supplementary material for: Serum FT3/FT4 ratio may indicate the degree of T3 compensation in congenital hypothyroidism
Source: PLoS One. 2026 Nov 11;21(11):e0361192. doi: 10.1371/journal.pone.0361192 (PMC13754123; doi:10.1371/journal.pone.0361192)
Supplement: Supplementary file 6 [file pone.0361192.s006.pdf]

Supplemental Fig. 2
Flowchart of the selection process of control group 2

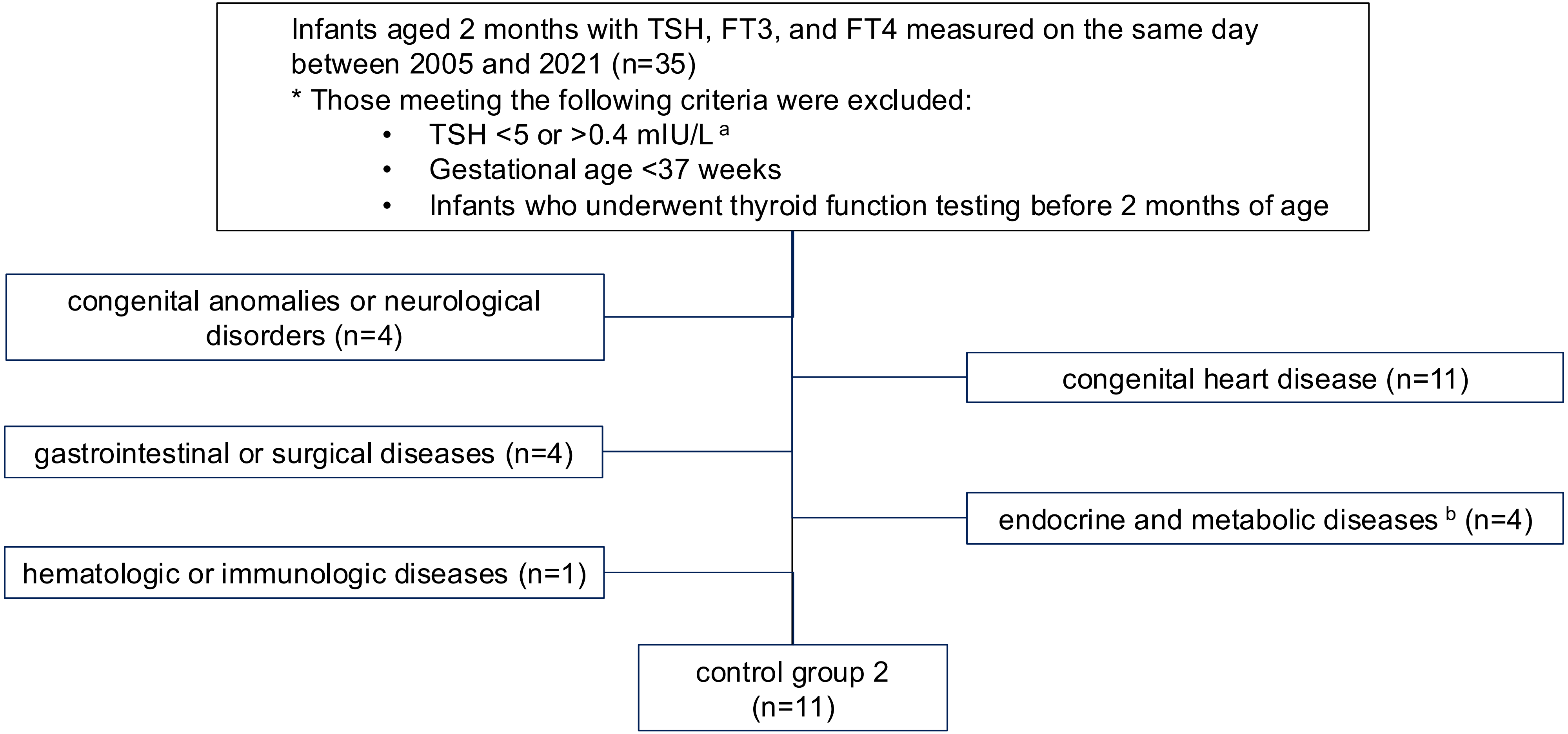

Of the 35 term infants aged 2 months with TSH, FT4, and FT3 measured on the same day, infants were excluded on the basis of the following criteria: serum TSH > 5.0 mIU/L or < 0.4 mIU/L, or the presence of congenital anomalies or neurological disorders, congenital heart disease, gastrointestinal or surgical diseases, endocrine and metabolic disease, and hematologic or immunologic diseases Therefore, 11 infants were selected as control group 2. a, Infants whose laboratory values were measured multiple times and met the criteria at least once were excluded; b, including infants born to mothers with Grave's disease; TSH, thyroid stimulating hormone; FT4, free thyroxine; FT3, free triiodothyronine
