# Supplementary material for: Serum FT3/FT4 ratio may indicate the degree of T3 compensation in congenital hypothyroidism
Source: PLoS One. 2026 Nov 11;21(11):e0361192. doi: 10.1371/journal.pone.0361192 (PMC13754123; doi:10.1371/journal.pone.0361192)
Supplement: Supplementary file 7 [file pone.0361192.s007.pdf]

Supplemental Fig. 3
Scatter diagram of serum TSH concentrations and FT3/FT4 ratios.

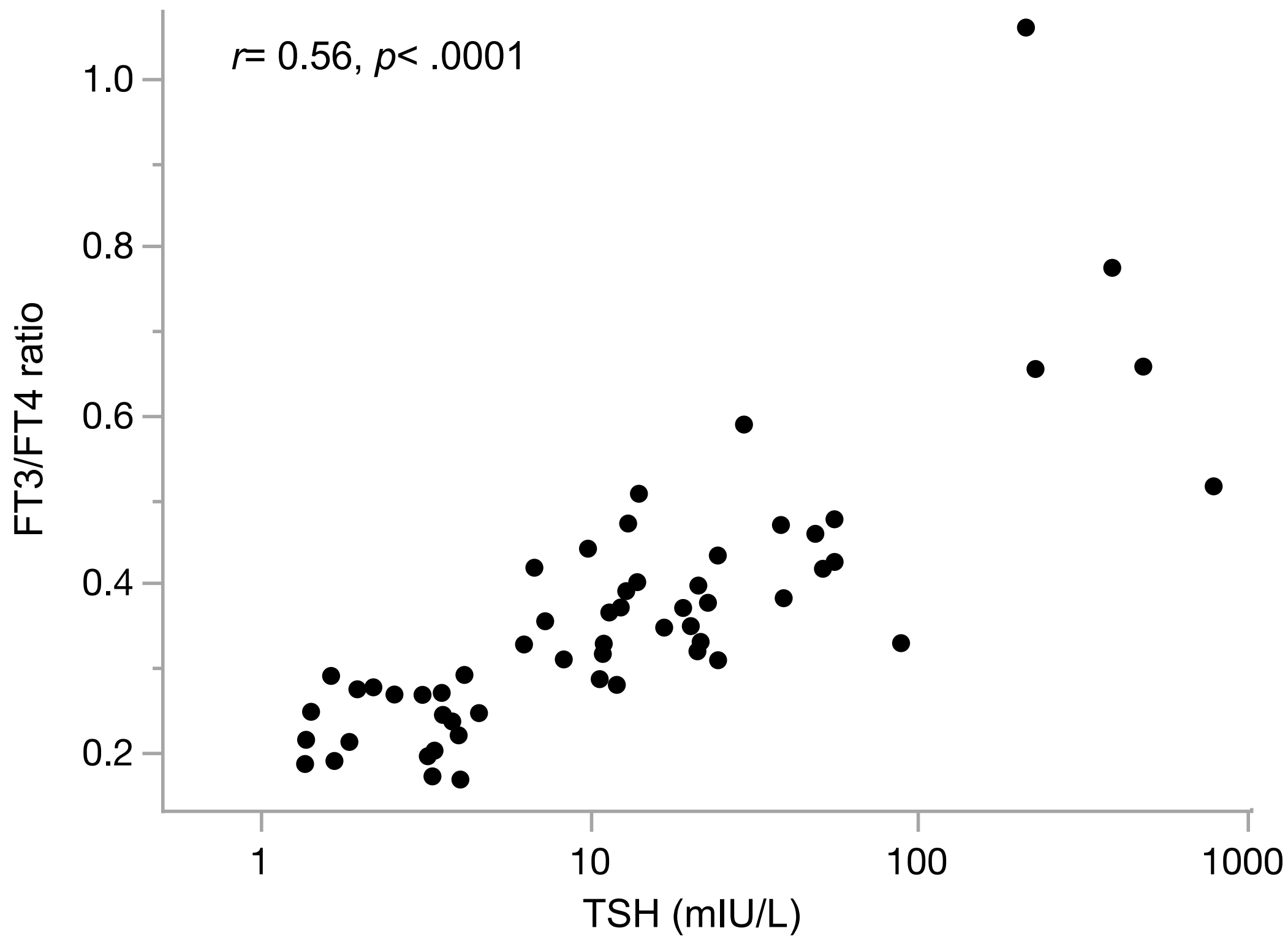

The correlation coefficient was r = 0.56 (p<0.0001), indicating a moderate correlation. TSH, thyroid stimulating hormone; FT3, free triiodothyronine; FT4, free thyroxine; FT3/FT4 ratio, ratio of free T3 to free T4
