# Supplementary material for: Serum FT3/FT4 ratio may indicate the degree of T3 compensation in congenital hypothyroidism
Source: PLoS One. 2026 Nov 11;21(11):e0361192. doi: 10.1371/journal.pone.0361192 (PMC13754123; doi:10.1371/journal.pone.0361192)
Supplement: Supplementary file 8 [file pone.0361192.s008.pdf]

Supplemental Fig. 4
Receiver operating characteristic (ROC) curve for serum FT3/FT4 ratio to predict CH*.

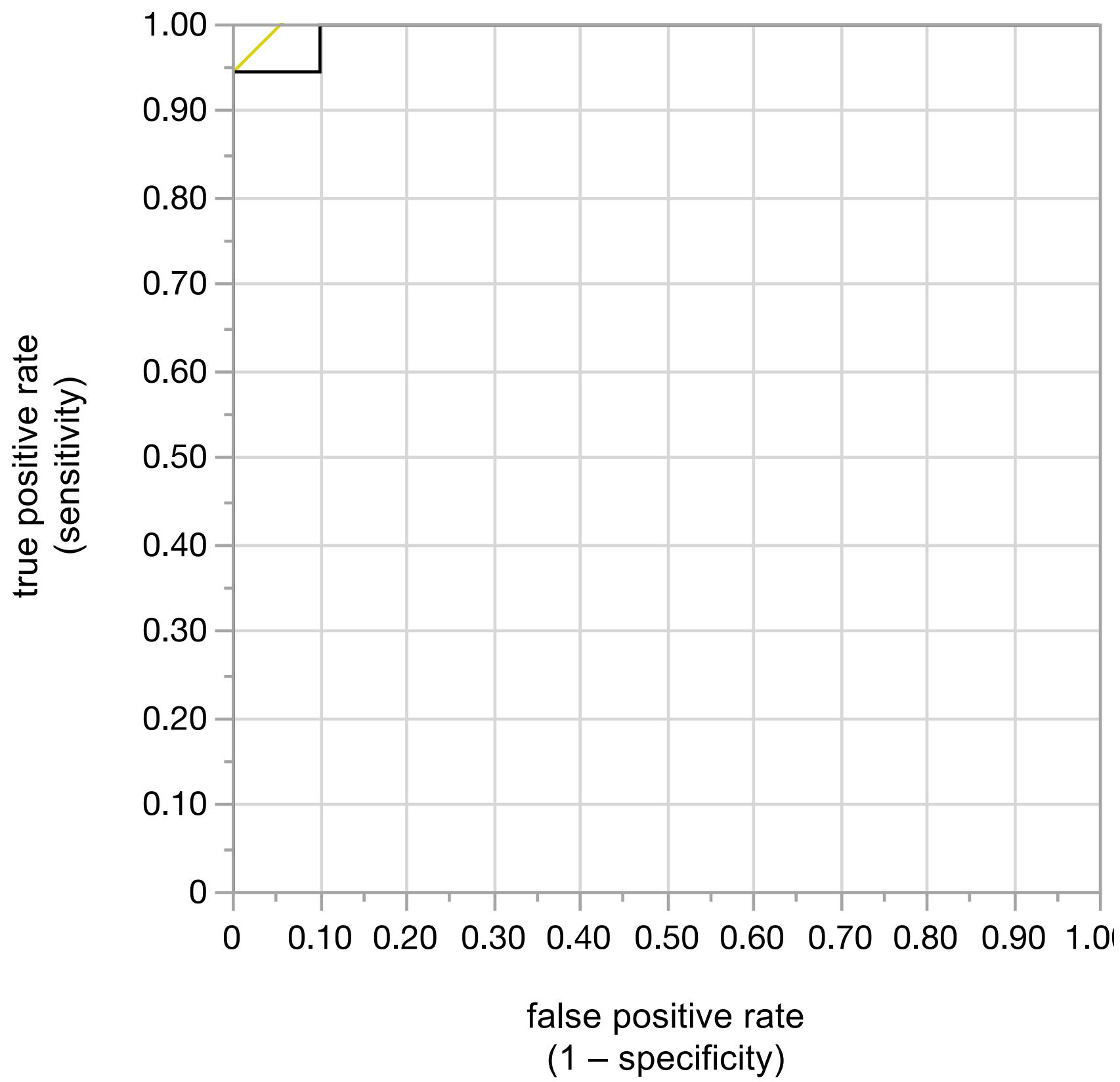

Area Under the Curve is 0.99
*; CH includes severe, moderate with LT4, and moderate with observation
