# Supplementary material for: Serum FT3/FT4 ratio may indicate the degree of T3 compensation in congenital hypothyroidism
Source: PLoS One. 2026 Nov 11;21(11):e0361192. doi: 10.1371/journal.pone.0361192 (PMC13754123; doi:10.1371/journal.pone.0361192)
Supplement: Supplementary file 9 [file pone.0361192.s009.pdf]

Supplemental Fig. 5
Changes in TSH concentrations at diagnosis and after 2 months in the moderate CH with observation group.

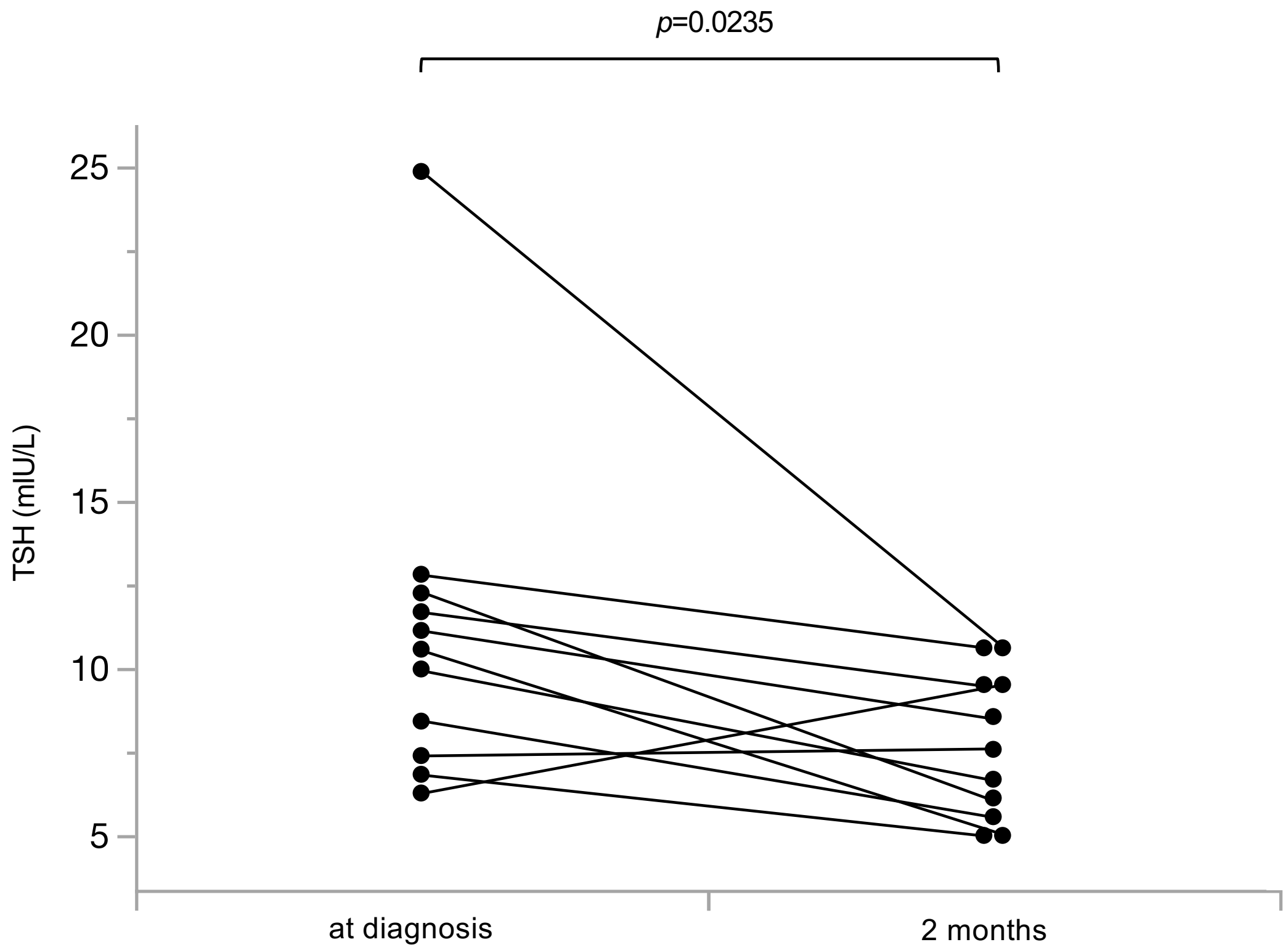

No treatment was administered, but TSH concentrations decreased over 2 months. CH, congenital hypothyroidism; TSH, thyroid stimulating hormone
