# Supplementary material for: Serum FT3/FT4 ratio may indicate the degree of T3 compensation in congenital hypothyroidism
Source: PLoS One. 2026 Nov 11;21(11):e0361192. doi: 10.1371/journal.pone.0361192 (PMC13754123; doi:10.1371/journal.pone.0361192)
Supplement: Supplementary file 11 [file pone.0361192.s011.docx]

Silvia Naitza, Ph.D

Academic Editor

*PLOS One*

Date July 2, 2026

Dear Academic Editor Dr. Silvia Naitza,

Subject: Submission of revised paper PONE-D-26-09218

We sincerely appreciate the editor and reviewers’ valuable and insightful comments on our manuscript. We have revised and improved our manuscript as suggested. In the following, we provide a point-to-point response to every question raised. We have highlighted portions of the manuscript that have been updated in response to the reviewers’ comments.

We have addressed the editorial points as follows.

In accordance with Reviewer #2’s comments, we have revised the analysis period in the original manuscript from 2005 to 2023 to 2005 to 2021. Consequently, the number of participants and the numerical values in the results have changed. However, the main statistical results and the interpretation of those results remain largely unchanged from the pre-revision version.

We hope the revised version is now suitable for publication and look forward to hearing from you in due course.

Sincerely,

Takeru Yamauchi

Program-Specific Assistant Professor

Kyoto University

” Point to Point Response to the Reviewers”

Manuscript ID: PONE-D-26-09218

*Reviewer #1:*

*In this study, the Authors investigated whether the serum FT3/FT4 ratio could be an indicator of congenital hypothyroidism (CH) severity, instead of serum TSH level. To this end, they analyzed 38 newborns with primary CH at diagnosis and 2 months after LT4 treatment or follow up, and a control group 1 aged 7-30 days (n=29) and a control group 2 aged 2 months (n=13). They found that serum FT3/FT4 ratio was elevated in CH infants before treatment compared with the control group 1, reflecting CH severity. They also found that 2 months after diagnosis, serum FT3/FT4 ratio decreased significantly in infants who started the replacement therapy, while no change was observed in CH infants with moderate severity of hypothyroidism who did not started treatment. The Authors concluded that a high serum FT3/FT4 ratio may be an indicator of hypothyroidism severity.*

*Minor observations*

***Comment1.***

*Although the aim of this study was to verify whether in primary CH serum FT3/FT4 ratio may be an indicator of severity instead of serum TSH, at diagnosis FT3/FT4 ratio showed a decreasing trend over the groups overlapping that observed for serum TSH (FIG.2 panel A and D). In addition, 2 months after treatment or follow up, while median TSH was significantly higher in moderate CH infants who did not start therapy than in all the other groups (indicating a high risk of hypothyroidism requiring treatment), in the same infants FT3/FT4 ratio did not show any difference with control group 2. These findings suggest that in primary CH serum TSH is the most sensitive biomarker of thyroid function and that FT3/FT4 ratio provides a similar trend of TSH but with a lower sensitivity. This concept should be clearly expressed in the Discussion. Similarly, in the Conclusion it should be underlined that while FT3/FT4 ratio may be useful in the diagnosis of central hypothyroidism (that cannot be diagnosed using TSH alone), it shows limited benefit in primary CH.*

[Response]

We appreciate the reviewer’s careful assessment and the opportunity to clarify this point. As the reviewer notes, our findings do not indicate that the serum FT3/FT4 ratio is superior to serum TSH for assessing primary congenital hypothyroidism. Rather, we interpret the serum FT3/FT4 ratio as a potential complementary marker, particularly in settings such as central hypothyroidism, in which serum TSH alone is not sufficient for diagnosis. We have rewritten the text to clarify this.

- Lines 71–74

<Original manuscript>

In summary, diagnosing hypothyroidism and assessing treatment efficacy based solely on serum TSH and FT4 levels has limitations. Consequently, it is imperative to identify an alternative to serum TSH that can more precisely evaluate the diagnosis and therapeutic outcomes of hypothyroidism.

<After revision>

Although conventional serum TSH and FT4 measurements are sufficient for the diagnosis and management of most cases of primary hypothyroidism and severe central hypothyroidism, some cases cannot be fully evaluated using these markers alone. Therefore, additional markers that complement serum TSH may help support a more comprehensive evaluation of hypothyroidism diagnosis and treatment response.

***Comment2.***

*At lines 233-236 it is written “serum TSH concentrations at 2 months in the moderate CH with observation group were significantly lower than those at diagnosis (at diagnosis, 10.95 (7.64–12.47) vs 2 months, 7.57 (5.69–9.75), p= .016. But in Supplemental Table 3 showing hormone levels at 2 months, the median (Q1, Q3) TSH in this group of infants is 8.06 (5.75-9.63) mIU/L. This point needs to be clarified.*

[Response]

We appreciate the reviewer’s careful observation. The discrepancy was due to a typographical error in Supplemental Table 3. In response to another reviewer’s comment regarding potential differences between assay kits, we reanalyzed the data by restricting the study population to individuals tested with the same kit between 2005 and 2021. Although the overall findings remained consistent, some numerical values, including TSH concentrations, changed after this reanalysis. We have revised the corresponding values in the main text accordingly.

***Comment3.***

*Line 259: To avoid any confusion between central and primary CH, change “In our patients diagnosed with CH..” with “In our patients diagnosed with primary CH.”*

<Response>

We thank the reviewer for this careful suggestion. To avoid confusion between central and primary congenital hypothyroidism, we have added the word “primary” as recommended.

**Reviewer #2: Major Comments**

**1. Overinterpretation of CNS thyroid hormone deficiency**

*The discussion repeatedly suggests that the FT3/FT4 ratio may reflect “thyroid hormone deficiency in the CNS.” While this is an intriguing hypothesis, the current study does not directly assess CNS thyroid hormone status, neurodevelopmental outcomes, or tissue-specific thyroid signaling. The conclusions should therefore be moderated. The current data support an association between the FT3/FT4 ratio and the biochemical severity of CH, but do not allow direct inference about CNS thyroid hormone sufficiency.*

<Response>

We appreciate the reviewer’s insightful comment. As the reviewer correctly notes, the present study did not directly assess thyroid hormone status in the central nervous system, neurodevelopmental outcomes, or tissue-specific thyroid hormone signaling. Therefore, the data do not allow direct conclusions regarding thyroid hormone deficiency or compensatory mechanisms within the central nervous system. We have removed this discussion from the main text. At the same time, the observation that the FT3/FT4 ratio is markedly increased in MCT8 deficiency, a condition characterized by thyroid hormone deficiency in the central nervous system, informed the hypothesis underlying the present study. We have revised the manuscript to ensure that this point is presented more cautiously and is not overinterpreted.

- Lines 272–282: We have removed this from the main text.
- Line 238: The following sentence has been added:

<Original manuscript>

Second, Oto et al. compared the FT3/FT4 ratio in patients…

<Revision>

A report by Oto et al. supports this interpretation. They compared the FT3/FT4 ratio in patients…

- Lines 291–299 have been changed as follows.

<Original manuscript>

Considering these findings, the extent of serum FT3/FT4 ratio elevation might indicate the degree of neuronal TH deficiency in both patients with primary hypothyroidism and those with CH. CH involves TH deficiency from birth; therefore, the CNS of patients with CH prior to treatment is also considered to be in a state of TH deficiency. A high serum FT3/FT4 ratio may reflect TH deficiency in the CNS. Furthermore, the decrease in the serum FT3/FT4 ratio with LT4 administration may be the result of suppressed conversion of T4 to T3 to avoid excess TH in the CNS. Moreover, a decrease in serum FT3 concentration with LT4 administration for TSH normalization may not indicate insufficient treatment but may indicate TH sufficiency in the CNS.

<Revision>

Taken together, these findings suggest that an increased serum FT3/FT4 ratio in patients with primary hypothyroidism, including CH, may reflect systemic TH deficiency beyond the hypothalamic–pituitary axis. Conversely, the decrease in the serum FT3/FT4 ratio after LT4 administration may reflect suppression of T4-to-T3 conversion to avoid systemic TH excess. Thus, a decrease in serum FT3 concentration during LT4 treatment aimed at TSH normalization may not necessarily indicate insufficient treatment; rather, it may indicate that circulating T4 exceeds systemic TH requirements.

- Lines 321–323

<Original manuscript>

Therefore, in patients with untreated hypothyroidism, the serum FT3/FT4 ratio may be considered an indicator of the degree of T3 compensation and possibly reflects TH deficiency in the CNS.

<Revision>

Therefore, in patients with untreated hypothyroidism, the serum FT3/FT4 ratio may be considered an indicator of the degree of T3 compensation.

**2. Definition of severity based solely on TSH**

*The study defines “severe” and “moderate” CH based solely on TSH thresholds. However, the central premise of the paper is that TSH may not adequately reflect hypothyroidism severity. This creates some conceptual circularity. The authors should discuss this limitation explicitly and consider whether FT4-based or combined criteria might better reflect disease severity.*

<Response>

We thank the reviewer for raising this important conceptual point. In response to this comment, we recognized that our original wording may have led readers to misinterpret the study objective. The primary aim of this study was to assess whether the serum FT3/FT4 ratio could serve as an indicator of hypothyroidism in untreated patients, rather than to demonstrate that the serum FT3/FT4 ratio is a better marker of disease severity than serum TSH. Because the present study does not provide sufficient data to support such a conclusion, further investigation will be required. We have revised the wording accordingly to avoid this potential misunderstanding.

- Lines 71-74

<Original manuscript>

In summary, diagnosing hypothyroidism and assessing treatment efficacy based solely on serum TSH and FT4 levels has limitations. Consequently, it is imperative to identify an alternative to serum TSH that can more precisely evaluate the diagnosis and therapeutic outcomes of hypothyroidism.

<Revision>

Although conventional serum TSH and FT4 measurements are sufficient for the diagnosis and management of most cases of primary hypothyroidism and severe central hypothyroidism, some cases cannot be fully evaluated using these markers alone. Therefore, additional markers that complement serum TSH may help support a more comprehensive evaluation of hypothyroidism diagnosis and treatment response.

**3. Potential confounding by age at diagnosis**

*There were significant differences in the day of diagnosis among groups. Because neonatal thyroid hormone physiology changes rapidly during the first weeks of life, age-related changes may independently influence FT3, FT4, and the FT3/FT4 ratio, regardless of CH severity. The authors should address this more thoroughly, ideally by:*

*o adjusting analyses for age at sampling,*

*o performing correlation analyses between age and FT3/FT4 ratio, or*

*o discussing this as a major limitation.*

<Response>

We appreciate the opportunity to address this issue in greater detail. Because the measurements were not obtained at the same age in all participants, adjustment for age at sampling was difficult. Therefore, we examined correlations between age and the serum FT3/FT4 ratio. No correlation was observed in the CH groups, and no correlation was observed between age and the serum FT3/FT4 ratio in Control group 1, which had no thyroid dysfunction (see the figure).

Based on these results, no age-related differences were observed in the control group or in the overall CH group, comprising the severe and moderate groups combined. Therefore, although this remains a limitation, the observed differences among the three groups are unlikely to be attributable to age alone. Accordingly, we concluded that the serum FT3/FT4 ratio during the neonatal period is minimally influenced by age and may reflect the severity of hypothyroidism.

**4. Control groups require clarification**

*The controls were not healthy infants but patients evaluated for prolonged jaundice, hemangiomas, or weight-gain concerns. Although thyroid disease was excluded, these conditions could potentially influence thyroid hormone metabolism. The authors should provide stronger justification for the suitability of these controls and discuss possible bias.*

<Response>

We appreciate the opportunity to clarify this point.

As the reviewer notes, the control group cannot be definitively classified as comprising healthy newborns. However, the control infants presented with mild jaundice that improved with phototherapy, relatively small hemangiomas, or poor weight gain without other findings, and no abnormalities were detected in standard thyroid function tests, including serum TSH and FT4. Although we cannot definitively exclude abnormalities in thyroid hormone metabolism, a substantial abnormality in the conversion of FT4 to FT3 would be expected to result in thyroid dysfunction, such as elevated serum TSH levels. Therefore, we considered that these infants were unlikely to have marked abnormalities in thyroid hormone metabolism and included them as control groups in this study. We have also noted this point in the Limitations section of the manuscript.

- Lines 314–316

<Original manuscript>

Finally, it should be noted that the two control groups were not completely disease-free. Owing to ethical limitations, we were unable to evaluate the patients’ thyroid function in detail.

<Revision>

Finally, the control groups cannot be definitively classified as healthy newborns. However, the control infants presented with mild jaundice that improved with phototherapy, relatively small hemangiomas, or poor weight gain without other findings, and no abnormalities were detected in standard thyroid function tests such as serum TSH and FT4. Although abnormalities in thyroid hormone metabolism cannot be completely excluded, a marked abnormality in FT4-to-FT3 conversion would be expected to cause thyroid dysfunction, such as elevated serum TSH. Therefore, these infants were considered unlikely to have marked abnormalities in thyroid hormone metabolism and were included as the control group in this study.

**5. Assay variability across the study period**

*Two different assay platforms were used over a long study period (2005–2021). Although TSH harmonization was performed, FT3 and FT4 harmonization could not be performed. Because the primary outcome is the FT3/FT4 ratio, assay-related variability remains a significant concern. The authors should:*

*o provide information on comparability between assays,*

*o indicate how many patients were measured using each platform,*

*o and consider sensitivity analyses restricted to one assay period if feasible.*

<Response>

We thank the reviewer for these important comments. As the reviewer points out, we cannot exclude the possibility of differences in the FT3/FT4 ratio between assay kits. Therefore, we reanalyzed the data by restricting the study population to individuals tested between 2005 and 2021, during which the same kit was used. These details have been added to the Patients subsection of the Methods and to the Results section. Please also refer to the attached files for further details.

- Lines 309–314: We have removed this from the main text.

Fourth, the examination kits we used varied over time. Although the harmonization coefficient was used to evaluate serum TSH data, no reliable coefficients were available for serum FT3 and FT4. Nevertheless, because the serum FT3/FT4 ratio is not a direct measurement, and serum FT3 and FT4 concentrations were measured using the same kit, the variability between examination kits was minimized.

**6. Statistical considerations**

*The sample size is small, especially after subgrouping. The manuscript would benefit from:*

*o effect size reporting,*

*o confidence intervals,*

*o and possibly regression analyses evaluating predictors of FT3/FT4 ratio.*

*In addition, the Bonferroni-corrected significance threshold is described as 0.83%, which should be expressed more clearly as p < 0.0083.*

<Response>

We appreciate these suggestions. We have added the standard error and 95% confidence intervals to Supplemental Tables 2 and 4. We have also revised the notation for the Bonferroni-corrected significance threshold to *p* < 0.0083.

*o and possibly regression analyses evaluating predictors of FT3/FT4 ratio.*

In addition, to further evaluate the discriminatory performance of the serum FT3/FT4 ratio, we added an ROC curve analysis to the Results section and Supplemental Figure. The following text will be incorporated before line 226 in the main text.

Receiver operating characteristic curve for the serum FT3/FT4 ratio at diagnosis

To determine the optimal cutoff value of the pretreatment serum FT3/FT4 ratio for distinguishing the CH groups (severe CH, moderate CH with LT4, and moderate CH with observation) from Control group 1, a receiver operating characteristic (ROC) curve was constructed (Supplemental Figure 4). This analysis identified a cutoff value of 0.30, with an area under the curve (AUC) of 0.99, sensitivity of 94.59%, and specificity of 100%.

**7. Clinical applicability remains uncertain**

*While the findings are interesting from a physiological standpoint, the practical clinical utility of the FT3/FT4 ratio remains unclear. The authors should elaborate on:*

*o how this ratio might alter clinical management,*

*o proposed cutoff values,*

*o and whether it adds value beyond FT4 alone in central hypothyroidism or mild CH.*

<Response>

We thank the reviewer for highlighting these important issues. The following text will be incorporated before line 300 in the main text.

The clinical utility of the serum FT3/FT4 ratio should be interpreted cautiously. Because the serum FT3/FT4 ratio correlated with serum TSH severity in this study, its usefulness in primary CH, for which severity can generally be assessed using serum TSH, is likely limited. In particular, the ratio is unlikely to alter clinical management in severe cases with clearly elevated serum TSH levels. In the moderate CH with observation group, serum TSH remained significantly elevated at 2 months, whereas the serum FT3/FT4 ratio did not differ significantly from that in the control group 2. These findings suggest that serum TSH may be a more sensitive marker of primary hypothyroidism. In contrast, the finding that the serum FT3/FT4 ratio increased in proportion to serum TSH elevation in neonatal hypothyroidism suggests that this index may have potential value in settings where serum TSH is less informative, such as central congenital hypothyroidism. Screening based on serum FT4 alone may miss mild-to-moderate central congenital hypothyroidism when the decrease in serum FT4 is slight. Therefore, the serum FT3/FT4 ratio may provide additional information for screening in such cases. However, the present study cannot definitively establish its utility for central congenital hypothyroidism or for mild primary CH, in which the need for treatment remains uncertain. Further studies are required to confirm this hypothesis. In addition, because the sample size of the present study was too small to establish definitive cutoff values, caution is warranted regarding their clinical application. If validated in large-scale datasets, such cutoff values could help inform therapeutic decision-making for mild central congenital hypothyroidism.

***Minor Comments*** *1. The manuscript would benefit from careful English editing for grammar and style. Several sentences in the Discussion are overly speculative and lengthy. Shortening and focusing the discussion would improve readability.
2. Please define all abbreviations consistently at first use (e.g., CNS, LT4).
3. The rationale for using FT3/FT4 ratio rather than FT3 alone should be emphasized more clearly in the Introduction.
4. Reference formatting appears inconsistent in several places and should be checked according to the journal style.*

[Response]

We appreciate the reviewer’s helpful suggestions. We have revised the manuscript to address each of these points.

*1. The manuscript would benefit from careful English editing for grammar and style. Several sentences in the Discussion are overly speculative and lengthy. Shortening and focusing the discussion would improve readability.*

Regarding the English language, grammar, and style, the manuscript has been thoroughly reviewed and revised by a professional English editing service to improve clarity and grammatical accuracy. All changes are tracked in the revised manuscript.

*2. Please define all abbreviations consistently at first use (e.g., CNS, LT4).*

We have ensured that abbreviations are now properly defined.

*3. The rationale for using FT3/FT4 ratio rather than FT3 alone should be emphasized more clearly in the Introduction.*

We have added the following text to the second paragraph of the Introduction.

- Line 85:

<Revision>

A relative elevation in serum FT3 concentration compared with serum FT4 concentration may therefore reflect compensatory changes in thyroid hormone regulation that are not fully captured by the absolute serum FT3 or FT4 concentration alone. In this context, evaluating the relationship between these two hormones may provide additional information.

***Overall Recommendation*** *This potentially valuable and novel study suggests that the FT3/FT4 ratio may reflect compensatory thyroid physiology in congenital hypothyroidism. I recommend a major revision before the manuscript can be considered for publication.*

[Response]

We thank the reviewer for the positive assessment of our work and for recommending major revision.
