# Supplementary material for: Serum FT3/FT4 ratio may indicate the degree of T3 compensation in congenital hypothyroidism
Source: PLoS One. 2026 Nov 11;21(11):e0361192. doi: 10.1371/journal.pone.0361192 (PMC13754123; doi:10.1371/journal.pone.0361192)
Supplement: Supplementary file 10 [file pone.0361192.s010.docx]

The study is novel in its evaluation of FT3/FT4 ratios before treatment in congenital hypothyroidism (CH), and the authors present biologically plausible mechanistic interpretations. This manuscript is, as a whole, well-organized and clinically interesting. However, some issues should be addressed to strengthen the scientific rigor and improve interpretability.

**Major Comments**

1. **Overinterpretation of CNS thyroid hormone deficiency**

The discussion repeatedly suggests that the FT3/FT4 ratio may reflect “thyroid hormone deficiency in the CNS.” While this is an intriguing hypothesis, the current study does not directly assess CNS thyroid hormone status, neurodevelopmental outcomes, or tissue-specific thyroid signaling. The conclusions should therefore be moderated. The current data support an association between the FT3/FT4 ratio and the biochemical severity of CH, but do not allow direct inference about CNS thyroid hormone sufficiency.

2. **Definition of severity based solely on TSH**

The study defines “severe” and “moderate” CH based solely on TSH thresholds. However, the central premise of the paper is that TSH may not adequately reflect hypothyroidism severity. This creates some conceptual circularity. The authors should discuss this limitation explicitly and consider whether FT4-based or combined criteria might better reflect disease severity.

3. **Potential confounding by age at diagnosis**

There were significant differences in the day of diagnosis among groups. Because neonatal thyroid hormone physiology changes rapidly during the first weeks of life, age-related changes may independently influence FT3, FT4, and the FT3/FT4 ratio, regardless of CH severity. The authors should address this more thoroughly, ideally by:

- adjusting analyses for age at sampling,
- performing correlation analyses between age and FT3/FT4 ratio, or
- discussing this as a major limitation.

4. **Control groups require clarification**

The controls were not healthy infants but patients evaluated for prolonged jaundice, hemangiomas, or weight-gain concerns. Although thyroid disease was excluded, these conditions could potentially influence thyroid hormone metabolism. The authors should provide stronger justification for the suitability of these controls and discuss possible bias.

5. **Assay variability across the study period**

Two different assay platforms were used over a long study period (2005–2021). Although TSH harmonization was performed, FT3 and FT4 harmonization could not be performed. Because the primary outcome is the FT3/FT4 ratio, assay-related variability remains a significant concern. The authors should:

- provide information on comparability between assays,
- indicate how many patients were measured using each platform,
- and consider sensitivity analyses restricted to one assay period if feasible.

6. **Statistical considerations**

The sample size is small, especially after subgrouping. The manuscript would benefit from:

- effect size reporting,
- confidence intervals,
- and possibly regression analyses evaluating predictors of FT3/FT4 ratio.

In addition, the Bonferroni-corrected significance threshold is described as 0.83%, which should be expressed more clearly as p < 0.0083.

7. **Clinical applicability remains uncertain**

While the findings are interesting from a physiological standpoint, the practical clinical utility of the FT3/FT4 ratio remains unclear. The authors should elaborate on:

- how this ratio might alter clinical management,
- proposed cutoff values,
- and whether it adds value beyond FT4 alone in central hypothyroidism or mild CH.

**Minor Comments**

1. The manuscript would benefit from careful English editing for grammar and style. Several sentences in the Discussion are overly speculative and lengthy. Shortening and focusing the discussion would improve readability.
2. Please define all abbreviations consistently at first use (e.g., CNS, LT4).
3. The rationale for using FT3/FT4 ratio rather than FT3 alone should be emphasized more clearly in the Introduction.
4. Reference formatting appears inconsistent in several places and should be checked according to the journal style.

**Overall Recommendation**

This potentially valuable and novel study suggests that the FT3/FT4 ratio may reflect compensatory thyroid physiology in congenital hypothyroidism. I recommend a **major revision** before the manuscript can be considered for publication.

Top of Form

Bottom of Form
