# Supplementary material for: Serum FT3/FT4 ratio may indicate the degree of T3 compensation in congenital hypothyroidism
Source: PLoS One. 2026 Nov 11;21(11):e0361192. doi: 10.1371/journal.pone.0361192 (PMC13754123; doi:10.1371/journal.pone.0361192)
Supplement: Supplementary file 13 [file pone.0361192.s013.docx]

I've read through the full manuscript. Here's my review.

**Overall Assessment**

This is a well-conceived, clinically relevant study addressing a real gap — how to assess hypothyroidism severity and treatment adequacy when TSH is uninformative (e.g., central hypothyroidism). The FT3/FT4 ratio is a plausible, low-cost, generalizable biomarker, and using congenital hypothyroidism (CH) as a "clean" natural experiment (defined onset, known treatment timing) is a smart study design choice. The core finding — ratio rises with severity, falls with LT4 treatment — is coherent with the deiodinase physiology discussed. However, there are several issues around internal consistency, statistical transparency, and language polish that should be addressed before submission.

**Major Issues**

**1. Internal contradiction in the Results (2-month FT3 comparison).** In the "Thyroid function parameters after 2 months" paragraph, one sentence states serum FT3 *differed significantly* between the moderate CH-with-TH group and control group 2, and the very next sentence states there were *no* significant differences in FT3 between any groups. These directly contradict each other and need to be resolved — likely a leftover edit.

**2. Abstract vs. Results numbers don't clearly map, and the "control group" framing in the abstract is potentially misleading.** The abstract reports "control group: 0.24 vs 2 months, 0.34" without a p-value, implying a paired within-group change. But control group 1 (n=20, at diagnosis timepoint) and control group 2 (n=11, at 2-month timepoint) are two *different, non-overlapping cohorts*, not the same infants followed longitudinally. Presenting them side-by-side as "before vs after" invites the reader to assume a paired comparison. Worth either rewording (e.g., "control group 1 vs control group 2") or explicitly flagging that these are separate cross-sectional controls.

**3. Small sample size and the ROC/AUC analysis.** An AUC of 0.99 with 94.6% sensitivity/100% specificity from a pool of 37 patients vs. 20 controls is very likely optimistic/overfit. This is somewhat addressed in the limitations paragraph, but the ROC result is stated quite confidently in the Results without a caveat at first mention — consider softening the framing there too, not just in Discussion.

**4. Multiple comparisons across many subgroups with modest n per cell (n=11–13).** The statistics section is appropriately rigorous (Bonferroni-adjusted α = 0.0083), but with cell sizes this small, non-significant results (e.g., "no significant difference detected") should be interpreted cautiously as likely underpowered rather than as evidence of equivalence — this distinction isn't always maintained in the Results/Discussion.

**Structural / Clarity Issues**

- The subheading "Comparison of the FT3/**F4** ratio and TSH..." is missing a "T" (should be FT3/FT4).
- Several sentences begin with "Whereas..." as if it's a standalone contrastive clause (e.g., "Whereas the remaining 20%...", "Whereas, serum FT3 concentrations did not differ..."). This isn't standard English usage — "whereas" needs to connect two clauses in one sentence, or should be replaced with "However" / "In contrast" when starting a new sentence.
- Typo: "mya" → "may" (Discussion, paragraph 1: "Elevated TSH in hypothyroidism mya also directly stimulate...").
- Typo: "primally CH" → likely "primary CH" (Discussion, paragraph 2).
- Typo: "withsevere thyroid hypoplasia" → missing space (Reference 13).
- Double period at end of limitations paragraph ("...included as the control group in this study..").
- Reference 19 author list has "Vitek F" — worth double-checking against the original citation (Pilo et al. 1990) for a possible transcription error in author names/initials.
- The Methods table is dense and hard to parse in the converted view, but that's a Word table formatting matter — worth double-checking that it renders cleanly as a proper Word table (not a compressed grid) in the final file, since some cells looked awkwardly narrow.

**Discussion-Level Suggestions**

- The claim that a *falling* FT3/FT4 ratio during LT4 treatment might reflect "appropriate suppression of T4-to-T3 conversion" rather than treatment inadequacy is an interesting reinterpretation of prior literature (refs 23–25), but it remains speculative since reverse T3 wasn't measured (rightly flagged as a limitation) — consider tempering the certainty of phrasing slightly in the paragraph itself, since the limitations section already does this well. Still, the body text reads a bit more assertively.
- The manuscript would benefit from one explicit sentence early in the Discussion restating the central clinical implication in plain terms (e.g., "in settings where TSH is unreliable, an elevated FT3/FT4 ratio could support a diagnosis of undertreated or untreated hypothyroidism") — right now the take-home message is present but distributed across several paragraphs.

**Minor/Optional**

- Consider whether "not rare condition" (Introduction) could be phrased more naturally, e.g., "an underrecognized rather than truly rare condition."
- The Funding/Competing Interests/Data Availability/Consent sections are clean and properly reported — no issues there.
- Figure legends are thorough and well-explained.
