# Supplementary material for: Serum FT3/FT4 ratio may indicate the degree of T3 compensation in congenital hypothyroidism
Source: PLoS One. 2026 Nov 11;21(11):e0361192. doi: 10.1371/journal.pone.0361192 (PMC13754123; doi:10.1371/journal.pone.0361192)
Supplement: Supplementary file 14 [file pone.0361192.s014.docx]

Silvia Naitza, Ph.D

Academic Editor

*PLOS One*

Date September 14, 2026

Dear Academic Editor Dr. Silvia Naitza,

Subject: Submission of revised paper PONE-D-26-09218

We sincerely appreciate the editor and reviewers’ valuable and insightful comments on our manuscript. We have revised and improved our manuscript as suggested. In the following, we provide a point-to-point response to every question raised. We have highlighted portions of the manuscript that have been updated in response to the reviewers’ comments.

Please note that we have attached the table under the filename “Table.”

We hope the revised version is now suitable for publication and look forward to hearing from you in due course.

Sincerely,

Takeru Yamauchi

Program-Specific Assistant Professor

Kyoto University

” Point to Point Response to the Reviewers”

Manuscript ID: PONE-D-26-09218

*Reviewer #2:*

*I've read through the full manuscript. Here's my review.*

***Overall Assessment***

*This is a well-conceived, clinically relevant study addressing a real gap — how to assess hypothyroidism severity and treatment adequacy when TSH is uninformative (e.g., central hypothyroidism). The FT3/FT4 ratio is a plausible, low-cost, generalizable biomarker, and using congenital hypothyroidism (CH) as a "clean" natural experiment (defined onset, known treatment timing) is a smart study design choice. The core finding — ratio rises with severity, falls with LT4 treatment — is coherent with the deiodinase physiology discussed. However, there are several issues around internal consistency, statistical transparency, and language polish that should be addressed before submission.*

We wish to express our appreciation to the reviewers for their insightful comments on our paper. The comments have helped us significantly improve the paper.

**Major Issues**

1. ***Internal contradiction in the Results (2-month FT3 comparison).*** *In the "Thyroid function parameters after 2 months" paragraph, one sentence states serum FT3 differed significantly between the moderate CH-with-TH group and control group 2, and the very next sentence states there were no significant differences in FT3 between any groups. These directly contradict each other and need to be resolved — likely a leftover edit.*

We appreciate the reviewer’s helpful suggestion. We have added the word “other” to the following sentence, as it was missing.

- Line 214

Before revision:
There were no statistically significant differences in serum FT3 concentrations between any of the groups.

After revision:
There were no statistically significant differences in serum FT3 concentrations between any of the **other** groups.

2. ***Abstract vs. Results numbers don't clearly map, and the "control group" framing in the abstract is potentially misleading.*** *The abstract reports "control group: 0.24 vs 2 months, 0.34" without a p-value, implying a paired within-group change. But control group 1 (n=20, at diagnosis timepoint) and control group 2 (n=11, at 2-month timepoint) are two different, non-overlapping cohorts, not the same infants followed longitudinally. Presenting them side-by-side as "before vs after" invites the reader to assume a paired comparison. Worth either rewording (e.g., "control group 1 vs control group 2") or explicitly flagging that these are separate cross-sectional controls.*

We appreciate the reviewer for the valuable comment. As the reviewer pointed out, the original wording gave the impression that comparisons were being made within each group. We considered stating the results in the abstract as “Control Group 1: 0.24” versus “Control Group 2: 0.34,” but were unable to do so due to the character limit. Since the key finding of this study is that a significant change in the FT3/FT4 ratio was observed before and after treatment in the same patients, whereas no such change was observed in the untreated group, we have removed the term “control” from the Results section of the abstract. However, as you pointed out, it is important to clearly convey to readers that the “control” groups consist of two non-overlapping cohorts of different ages; therefore, we have revised the “Methods” section of the abstract as follows:

- Line 44

Before revision:

Serum TSH, FT4, FT3, and FT3/FT4 ratios were compared between each group and the control group at diagnosis and 2 months after treatment.

After revision:

Serum TSH, FT4, FT3, and FT3/FT4 ratios were compared between each group and the **two control groups of different ages** at diagnosis and 2 months after treatment.

- Lines 49-50

Before revision
control group: 0.24 vs 2 months, 0.34.

After revision:
**Deleted**

3. ***Small sample size and the ROC/AUC analysis.*** *An AUC of 0.99 with 94.6% sensitivity/100% specificity from a pool of 37 patients vs. 20 controls is very likely optimistic/overfit. This is somewhat addressed in the limitations paragraph, but the ROC result is stated quite confidently in the Results without a caveat at first mention — consider softening the framing there too, not just in Discussion.*

We thank the reviewer for the important suggestion. As the reviewer mentioned, the small sample size imposes limitations on the analysis and interpretation, so we have revised the wording in the “Results” section as follows.

- Lines 233-238

Before revision:

To determine the optimal cutoff value of the pretreatment serum FT3/FT4 ratio for distinguishing the CH groups (severe CH, moderate CH with TH, and moderate CH with observation) from Control group 1, a receiver operating characteristic (ROC) curve was constructed (Supplemental Figure 4). This analysis identified a cutoff value of 0.30, with an area under the curve (AUC) of 0.99, sensitivity of 94.59%, and specificity of 100% (Supplemental Figure 4).

After revision:

**To explore the potential cutoff value** of the pretreatment serum FT3/FT4 ratio for distinguishing the CH groups (severe CH, moderate CH with TH, and moderate CH with observation) from Control group 1, a receiver operating characteristic (ROC) curve was constructed (Supplemental Figure 4). This analysis **suggested a tentative cutoff value** of 0.30, with an area under the curve (AUC) of 0.99, sensitivity of 94.59%, and specificity of 100% (Supplemental Figure 4).

4. ***Multiple comparisons across many subgroups with modest n per cell (n=11–13).*** *The statistics section is appropriately rigorous (Bonferroni-adjusted α = 0.0083), but with cell sizes this small, non-significant results (e.g., "no significant difference detected") should be interpreted cautiously as likely underpowered rather than as evidence of equivalence — this distinction isn't always maintained in the Results/Discussion.*

We appreciate the reviewer’s insightful suggestion regarding this important point. To help readers correctly recognize the limitations of our study, we have appended the following text to the discussion on sample size within the study limitations section.

- Lines 318-319

Before revision

This study has several limitations. First, it was a retrospective study performed at a single hospital, which limited the number of patients available for analysis.

After revision

This study has several limitations. First, it was a retrospective study performed at a single hospital, which limited the number of patients available for analysis. **Particularly regarding results where no significant difference was observed in multiple comparisons, the reason may not only be that there is no difference between the two groups, but there also remains the possibility that the difference could not be detected due to an insufficient sample size.**

**Structural / Clarity Issues**

- *The subheading "Comparison of the FT3/****F4*** *ratio and TSH..." is missing a "T" (should be FT3/FT4).*

We thank the reviewer very much for taking the time to carefully review this matter as well. This error has been corrected in accordance with the reviewer's comment.

- *Several sentences begin with "Whereas..." as if it's a standalone contrastive clause (e.g., "Whereas the remaining 20%...", "Whereas, serum FT3 concentrations did not differ..."). This isn't standard English usage — "whereas" needs to connect two clauses in one sentence, or should be replaced with "However" / "In contrast" when starting a new sentence.*

We appreciate the reviewer’s feedback. The paper has been edited and rewritten by an experienced scientific editor, who has improved the grammar and stylistic expression of the paper.

- *Typo: "mya" → "may" (Discussion, paragraph 1: "Elevated TSH in hypothyroidism mya also directly stimulate...").*
- *Typo: "primally CH" → likely "primary CH" (Discussion, paragraph 2).*
- *Typo: "withsevere thyroid hypoplasia" → missing space (Reference 13).*
- *Double period at end of limitations paragraph ("...included as the control group in this study..").*
- *Reference 19 author list has "Vitek F" — worth double-checking against the original citation (Pilo et al. 1990) for a possible transcription error in author names/initials.*

We thank the reviewer very much for taking the time to carefully review these matters as well. These errors have been corrected in accordance with the reviewer's comment.

- *The Methods table is dense and hard to parse in the converted view, but that's a Word table formatting matter — worth double-checking that it renders cleanly as a proper Word table (not a compressed grid) in the final file, since some cells looked awkwardly narrow.*

In accordance with the reviewer's comment, we have revised the table and updated it to match the one in the attachment.

**Discussion-Level Suggestions**

- The claim that a *falling* FT3/FT4 ratio during LT4 treatment might reflect "appropriate suppression of T4-to-T3 conversion" rather than treatment inadequacy is an interesting reinterpretation of prior literature (refs 23–25), but it remains speculative since reverse T3 wasn't measured (rightly flagged as a limitation) — consider tempering the certainty of phrasing slightly in the paragraph itself, since the limitations section already does this well. Still, the body text reads a bit more assertively.

We appreciate the reviewer for pointing that out. We have revised the wording in the text to avoid giving the impression that it is too assertive.

- Lines 283-297

Before revision:
A report by Oto et al. supports this interpretation. They compared the FT3/FT4 ratio in patients with congenital hypothyroidism due to thyroid aplasia or hypoplasia who were treated with LT4, for whom serum TSH concentration appeared to be used as the indicator for LT4 adjustment, with that in patients with central hypothyroidism who were also treated with LT4, for whom serum FT4 concentration appeared to be used as the indicator for LT4 adjustment ^13^. Patients with central hypothyroidism had a higher FT3/FT4 ratio during treatment. Accordingly, a decrease in the serum FT3/FT4 ratio during LT4 administration, when TSH normalization is used as the treatment indicator may suggest LT4 overtreatment.
Taken together, these findings suggest that an increased serum FT3/FT4 ratio in patients with primary hypothyroidism, including CH, may reflect systemic TH deficiency beyond the hypothalamic–pituitary axis. Conversely, the decrease in the serum FT3/FT4 ratio after LT4 administration may reflect suppression of T4-to-T3 conversion to avoid systemic TH excess. Thus, a decrease in serum FT3 concentration during LT4 treatment aimed at TSH normalization may not necessarily indicate insufficient treatment; rather, it may indicate that circulating T4 exceeds systemic TH requirements.

After revision:

A report by Oto et al. **may** support this interpretation. They compared the FT3/FT4 ratio in patients with congenital hypothyroidism due to thyroid aplasia or hypoplasia who were treated with LT4, for whom serum TSH concentration appeared to be used as the indicator for LT4 adjustment, with that in patients with central hypothyroidism who were also treated with LT4, for whom serum FT4 concentration appeared to be used as the indicator for LT4 adjustment ^13^. Patients with central hypothyroidism had a higher FT3/FT4 ratio during treatment. Accordingly, a decrease in the serum FT3/FT4 ratio during LT4 administration, when TSH normalization is used as the treatment indicator **might** suggest LT4 overtreatment.

Taken together, these findings **could** suggest that an increased serum FT3/FT4 ratio in patients with primary hypothyroidism, including CH, may reflect systemic TH deficiency beyond the hypothalamic–pituitary axis. Conversely, the decrease in the serum FT3/FT4 ratio after LT4 administration **might** reflect **potentially** suppression of T4-to-T3 conversion to avoid systemic TH excess. Thus, a decrease in serum FT3 concentration during LT4 treatment aimed at TSH normalization may not necessarily indicate insufficient treatment; rather, it **could potentially** indicate that circulating T4 exceeds systemic TH requirements.

- The manuscript would benefit from one explicit sentence early in the Discussion restating the central clinical implication in plain terms (e.g., "in settings where TSH is unreliable, an elevated FT3/FT4 ratio could support a diagnosis of undertreated or untreated hypothyroidism") — right now the take-home message is present but distributed across several paragraphs.

We appreciate the reviewer's comment on this point. We have added the following text to line 257 of the first paragraph of the discussion section.

　　　After revision:

**The findings of this study suggest that in settings where serum TSH is unreliable, an elevated FT3/FT4 ratio could support a diagnosis of undertreated or untreated hypothyroidism.** **Specifically,** these results **imply** that under TH-deficient conditions, increased deiodinase activity and enhanced T4-to-T3 conversion may help to compensate for T3 deficiency.

**Minor/Optional**

- *Consider whether "not rare condition" (Introduction) could be phrased more naturally, e.g., "an underrecognized rather than truly rare condition."*

We appreciate for your suggestion. We have revised the text as you suggested.

- *The Funding/Competing Interests/Data Availability/Consent sections are clean and properly reported — no issues there.*
- *Figure legends are thorough and well-explained.*

We thank the reviewer very much for taking the time to carefully review these matters as well.
