# Supplementary material for: Serum FT3/FT4 ratio may indicate the degree of T3 compensation in congenital hypothyroidism
Source: PLoS One. 2026 Nov 11;21(11):e0361192. doi: 10.1371/journal.pone.0361192 (PMC13754123; doi:10.1371/journal.pone.0361192)
Supplement: Supplementary file 15 [file pone.0361192.s015.docx]

**Review: "Serum FT3/FT4 ratio may indicate the degree of T3 compensation in congenital hypothyroidism"**

**Summary**

The authors retrospectively studied neonates with primary congenital hypothyroidism (CH) at a single center. In this study, they argue that the ratio reflects systemic T3 compensation and could help diagnose congenital hypothyroidism, where TSH is uninformative, such as central hypothyroidism.

The question is reasonable. The writing is clear, and the authors are commendably cautious in places. However, the central conclusion extends well beyond what the data support, and several issues warrant consideration.

**Minor concerns**

- **Treatment dose and timing:** LT4 doses differed markedly, and time on treatment, adherence, and the exact timing of blood draws relative to LT4 dosing should be described. Mean dose of levothyroxine should be mentioned in methodology.
- **Assay changes over 16 years:** Only one assay platform is named, but data span 2005–2021. Were the same Elecsys generations used throughout? Harmonization is described for TSH only.
- **Unit and scale of the ratio:** Please state units (pmol/L ÷ pmol/L, presumably) so the ratio and cutoff can be reproduced elsewhere.
- **Inclusion criteria:** "TSH ≥ 5 mIU/L" defines CH, yet group classification cites the Japanese guideline. Clarify how FT4 factored in, since many CH definitions require low FT4 or rely on TSH alone, and whether any patients had transient CH.
- **Language:** Numerous sentence fragments are beginning with "Whereas," and tense/grammar inconsistencies (e.g., "may also directly stimulate").
- **Overall recommendation:**

The observation that FT3/FT4 is elevated in untreated neonatal primary CH is plausible and of some interest, and the pre-treatment data fill a gap. But the paper's headline interpretations (T3 compensation, utility in central hypothyroidism, overtreatment signal, a validated cutoff) go beyond what a small, single-center, TSH-anchored, age-confounded dataset can show. With a narrower claim, stronger analysis of incremental value over FT4, and age-appropriate controls, this could be a useful, modest contribution.
