# Supplementary material for: Serum FT3/FT4 ratio may indicate the degree of T3 compensation in congenital hypothyroidism
Source: PLoS One. 2026 Nov 11;21(11):e0361192. doi: 10.1371/journal.pone.0361192 (PMC13754123; doi:10.1371/journal.pone.0361192)
Supplement: Peer review history

# Peer review: Decision Letter 0

**DOI:** 10.1371/journal.pone.0361192.r001

**Authors:** Silvia Naitza

20 May 2026

PONE-D-26-09218

Serum FT3/FT4 ratio may indicate the degree of T3 compensation in congenital hypothyroidism

PLOS One

Dear Dr. Yamauchi,

Thank you for submitting your manuscript to PLOS ONE. After careful consideration, we feel that it has merit but does not fully meet PLOS ONE’s publication criteria as it currently stands. Therefore, we invite you to submit a revised version of the manuscript that addresses the points raised during the review process.

In particular, both reviews question whether the FT3/FT4 ratio provides clinically meaningful information beyond TSH in primary congenital hypothyroidism and recommend a more cautious interpretation of the findings. Therefore, the conclusions should be more rigorously supported from a methodological perspective.

Overall, the manuscript would benefit from a more cautious and clinically focused discussion, a clearer acknowledgment of methodological limitations, and further clarification of how the proposed biomarker could meaningfully influence clinical management.

Please submit your revised manuscript by Jul 04 2026 11:59PM. If you will need more time than this to complete your revisions, please reply to this message or contact the journal office at plosone@plos.org. When you're ready to submit your revision, log on to https://www.editorialmanager.com/pone/ and select the 'Submissions Needing Revision' folder to locate your manuscript file.

Please include the following items when submitting your revised manuscript:

- A letter that responds to each point raised by the academic editor and reviewer(s). You should upload this letter as a separate file labeled 'Response to Reviewers'.
- A marked-up copy of your manuscript that highlights changes made to the original version. You should upload this as a separate file labeled 'Revised Manuscript with Track Changes'.
- An unmarked version of your revised paper without tracked changes. You should upload this as a separate file labeled 'Manuscript'.

If you would like to make changes to your financial disclosure, please include your updated statement in your cover letter. Guidelines for resubmitting your figure files are available below the reviewer comments at the end of this letter.

If applicable, we recommend that you deposit your laboratory protocols in protocols.io to enhance the reproducibility of your results. Protocols.io assigns your protocol its own identifier (DOI) so that it can be cited independently in the future. For instructions see: https://journals.plos.org/plosone/s/submission-guidelines#loc-laboratory-protocols. Additionally, PLOS ONE offers an option for publishing peer-reviewed Lab Protocol articles, which describe protocols hosted on protocols.io. Read more information on sharing protocols at https://plos.org/protocols?utm_medium=editorial-email&utm_source=authorletters&utm_campaign=protocols.

As the corresponding author, your ORCID iD is verified in the submission system and will appear in the published article. PLOS supports the use of ORCID, and we encourage all coauthors to register for an ORCID iD and use it as well. Please encourage your coauthors to verify their ORCID iD within the submission system before final acceptance, as unverified ORCID iDs will not appear in the published article. Only the individual author can complete the verification step; PLOS staff cannot verify ORCID iDs on behalf of authors.

We look forward to receiving your revised manuscript.

Kind regards,

Silvia Naitza, Ph.D

Academic Editor

PLOS One

Journal Requirements:

When submitting your revision, we need you to address these additional requirements.

1. Please ensure that your manuscript meets PLOS ONE's style requirements, including those for file naming. The PLOS ONE style templates can be found at

https://journals.plos.org/plosone/s/file?id=wjVg/PLOSOne_formatting_sample_main_body.pdf and

https://journals.plos.org/plosone/s/file?id=ba62/PLOSOne_formatting_sample_title_authors_affiliations.pdf

2. When completing the data availability statement of the submission form, you indicated that you will make your data available on acceptance. We strongly recommend all authors decide on a data sharing plan before acceptance, as the process can be lengthy and hold up publication timelines. Please note that, though access restrictions are acceptable now, your entire data will need to be made freely accessible if your manuscript is accepted for publication. This policy applies to all data except where public deposition would breach compliance with the protocol approved by your research ethics board. If you are unable to adhere to our open data policy, please kindly revise your statement to explain your reasoning and we will seek the editor's input on an exemption. Please be assured that, once you have provided your new statement, the assessment of your exemption will not hold up the peer review process.

3. Please include captions for your Supporting Information files at the end of your manuscript, and update any in-text citations to match accordingly. Please see our Supporting Information guidelines for more information: http://journals.plos.org/plosone/s/supporting-information.

4. If the reviewer comments include a recommendation to cite specific previously published works, please review and evaluate these publications to determine whether they are relevant and should be cited. There is no requirement to cite these works unless the editor has indicated otherwise.

**Additional Editor Comments:**

Dear Dr. Yamauchi,

Thank you for submitting your manuscript, “Serum FT3/FT4 ratio may indicate the degree of T3 compensation in congenital hypothyroidism” to PLOS One. The paper has now been evaluated by two external reviewers.

The reviewers recognize the potential contribution of your work, however, they have identified substantive issues that must be addressed before the manuscript can be considered further. One reviewer recommends minor revision, while another recommends major revision. After weighing these assessments and the editorial standards of the journal, I am requesting a major revision.

Please provide a thoroughly revised manuscript and a detailed, point-by-point response to each reviewer comment. In your response letter, include:

• A list of all changes made, referencing line numbers or sections.

• A clear rationale where you disagree with a suggestion, supported by evidence or citations.

• Any additional analyses, data, or methodological clarifications required by the reviewers.

Please note that a request for major revisions does not guarantee acceptance, as your revised manuscript may be sent back to the original reviewers for further evaluation.

We appreciate your contribution to PLOS One and look forward to your revision.

Best regards,

Silvia Naitza

[Note: HTML markup is below. Please do not edit.]

Reviewers' comments:

Reviewer's Responses to Questions

**Comments to the Author**

1. Is the manuscript technically sound, and do the data support the conclusions?

Reviewer #1: Yes

Reviewer #2: Partly

**********

2. Has the statistical analysis been performed appropriately and rigorously?

Reviewer #1: Yes

Reviewer #2: No

**********

3. Have the authors made all data underlying the findings in their manuscript fully available?

The PLOS Data policy

Reviewer #1: Yes

Reviewer #2: No

**********

4. Is the manuscript presented in an intelligible fashion and written in standard English?

Reviewer #1: Yes

Reviewer #2: No

**********

Reviewer #1: In this study, the Authors investigated whether the serum FT3/FT4 ratio could be an indicator of congenital hypothyroidism (CH) severity, instead of serum TSH level. To this end, they analyzed 38 newborns with primary CH at diagnosis and 2 months after LT4 treatment or follow up, and a control group 1 aged 7-30 days (n=29) and a control group 2 aged 2 months (n=13). They found that serum FT3/FT4 ratio was elevated in CH infants before treatment compared with the control group 1, reflecting CH severity. They also found that 2 months after diagnosis, serum FT3/FT4 ratio decreased significantly in infants who started the replacement therapy, while no change was observed in CH infants with moderate severity of hypothyroidism who did not started treatment. The Authors concluded that a high serum FT3/FT4 ratio may be an indicator of hypothyroidism severity.

Minor observations

Although the aim of this study was to verify whether in primary CH serum FT3/FT4 ratio may be an indicator of severity instead of serum TSH, at diagnosis FT3/FT4 ratio showed a decreasing trend over the groups overlapping that observed for serum TSH (FIG.2 panel A and D). In addition, 2 months after treatment or follow up, while median TSH was significantly higher in moderate CH infants who did not start therapy than in all the other groups (indicating a high risk of hypothyroidism requiring treatment), in the same infants FT3/FT4 ratio did not show any difference with control group 2. These findings suggest that in primary CH serum TSH is the most sensitive biomarker of thyroid function and that FT3/FT4 ratio provides a similar trend of TSH but with a lower sensitivity. This concept should be clearly expressed in the Discussion. Similarly, in the Conclusion it should be underlined that while FT3/FT4 ratio may be useful in the diagnosis of central hypothyroidism (that cannot be diagnosed using TSH alone), it shows limited benefit in primary CH.

At lines 233-236 it is written “serum TSH concentrations at 2 months in the moderate CH with observation group were significantly lower than those at diagnosis (at diagnosis, 10.95 (7.64–12.47) vs 2 months, 7.57 (5.69–9.75), p= .016. But in Supplemental Table 3 showing hormone levels at 2 months, the median (Q1, Q3) TSH in this group of infants is 8.06 (5.75-9.63) mIU/L. This point needs to be clarified.

Line 259: To avoid any confusion between central and primary CH, change “In our patients diagnosed with CH..” with “In our patients diagnosed with primary CH.”

Reviewer #2: Major Comments

1. Overinterpretation of CNS thyroid hormone deficiency

The discussion repeatedly suggests that the FT3/FT4 ratio may reflect “thyroid hormone deficiency in the CNS.” While this is an intriguing hypothesis, the current study does not directly assess CNS thyroid hormone status, neurodevelopmental outcomes, or tissue-specific thyroid signaling. The conclusions should therefore be moderated. The current data support an association between the FT3/FT4 ratio and the biochemical severity of CH, but do not allow direct inference about CNS thyroid hormone sufficiency.

2. Definition of severity based solely on TSH

The study defines “severe” and “moderate” CH based solely on TSH thresholds. However, the central premise of the paper is that TSH may not adequately reflect hypothyroidism severity. This creates some conceptual circularity. The authors should discuss this limitation explicitly and consider whether FT4-based or combined criteria might better reflect disease severity.

3. Potential confounding by age at diagnosis

There were significant differences in the day of diagnosis among groups. Because neonatal thyroid hormone physiology changes rapidly during the first weeks of life, age-related changes may independently influence FT3, FT4, and the FT3/FT4 ratio, regardless of CH severity. The authors should address this more thoroughly, ideally by:

o adjusting analyses for age at sampling,

o performing correlation analyses between age and FT3/FT4 ratio, or

o discussing this as a major limitation.

4. Control groups require clarification

The controls were not healthy infants but patients evaluated for prolonged jaundice, hemangiomas, or weight-gain concerns. Although thyroid disease was excluded, these conditions could potentially influence thyroid hormone metabolism. The authors should provide stronger justification for the suitability of these controls and discuss possible bias.

5. Assay variability across the study period

Two different assay platforms were used over a long study period (2005–2021). Although TSH harmonization was performed, FT3 and FT4 harmonization could not be performed. Because the primary outcome is the FT3/FT4 ratio, assay-related variability remains a significant concern. The authors should:

o provide information on comparability between assays,

o indicate how many patients were measured using each platform,

o and consider sensitivity analyses restricted to one assay period if feasible.

6. Statistical considerations

The sample size is small, especially after subgrouping. The manuscript would benefit from:

o effect size reporting,

o confidence intervals,

o and possibly regression analyses evaluating predictors of FT3/FT4 ratio.

In addition, the Bonferroni-corrected significance threshold is described as 0.83%, which should be expressed more clearly as p < 0.0083.

7. Clinical applicability remains uncertain

While the findings are interesting from a physiological standpoint, the practical clinical utility of the FT3/FT4 ratio remains unclear. The authors should elaborate on:

o how this ratio might alter clinical management,

o proposed cutoff values,

o and whether it adds value beyond FT4 alone in central hypothyroidism or mild CH.

Minor Comments

1. The manuscript would benefit from careful English editing for grammar and style. Several sentences in the Discussion are overly speculative and lengthy. Shortening and focusing the discussion would improve readability.

2. Please define all abbreviations consistently at first use (e.g., CNS, LT4).

3. The rationale for using FT3/FT4 ratio rather than FT3 alone should be emphasized more clearly in the Introduction.

4. Reference formatting appears inconsistent in several places and should be checked according to the journal style.

Overall Recommendation

This potentially valuable and novel study suggests that the FT3/FT4 ratio may reflect compensatory thyroid physiology in congenital hypothyroidism. I recommend a major revision before the manuscript can be considered for publication.

**********

what does this mean?). If published, this will include your full peer review and any attached files.

If you choose “no”, your identity will remain anonymous but your review may still be made public.

**Do you want your identity to be public for this peer review?**  For information about this choice, including consent withdrawal, please see our Privacy Policy

Reviewer #1: No

Reviewer #2: **Yes:**  Murshed Ahamed Khan

**********

[NOTE: If reviewer comments were submitted as an attachment file, they will be attached to this email and accessible via the submission site. Please log into your account, locate the manuscript record, and check for the action link "View Attachments". If this link does not appear, there are no attachment files.]

To ensure your figures meet our technical requirements, please review our figure guidelines: https://journals.plos.org/plosone/s/figures

You may also use PLOS’s free figure tool, NAAS, to help you prepare publication quality figures: https://journals.plos.org/plosone/s/figures#loc-tools-for-figure-preparation.

NAAS will assess whether your figures meet our technical requirements by comparing each figure against our figure specifications.

Attachment Submitted filename: Reviewer Comments, PONE-D-26-09218.docx

# Author comment: Author response to Decision Letter 1

**DOI:** 10.1371/journal.pone.0361192.r002

22 Jul 2026

We have revised all figures and supporting information in conjunction with the revision of the manuscript.

Attachment Submitted filename: Yamauchi_Response_to_Reviewer.docx

# Peer review: Decision Letter 1

**DOI:** 10.1371/journal.pone.0361192.r003

**Authors:** Silvia Naitza

12 Aug 2026

plosone@plos.org. When you're ready to submit your revision, log on to https://www.editorialmanager.com/pone/ and select the 'Submissions Needing Revision' folder to locate your manuscript file.

- A letter that responds to each point raised by the academic editor and reviewer(s). You should upload this letter as a separate file labeled 'Response to Reviewers'.
- A marked-up copy of your manuscript that highlights changes made to the original version. You should upload this as a separate file labeled 'Revised Manuscript with Track Changes'.
- An unmarked version of your revised paper without tracked changes. You should upload this as a separate file labeled 'Manuscript'.

If you would like to make changes to your financial disclosure, please include your updated statement in your cover letter. Guidelines for resubmitting your figure files are available below the reviewer comments at the end of this letter.

If applicable, we recommend that you deposit your laboratory protocols in protocols.io to enhance the reproducibility of your results. Protocols.io assigns your protocol its own identifier (DOI) so that it can be cited independently in the future. For instructions see: https://journals.plos.org/plosone/s/submission-guidelines#loc-laboratory-protocols. Additionally, PLOS ONE offers an option for publishing peer-reviewed Lab Protocol articles, which describe protocols hosted on protocols.io. Read more information on sharing protocols at https://plos.org/protocols?utm_medium=editorial-email&utm_source=authorletters&utm_campaign=protocols.

As the corresponding author, your ORCID iD is verified in the submission system and will appear in the published article. PLOS supports the use of ORCID, and we encourage all coauthors to register for an ORCID iD and use it as well. Please encourage your coauthors to verify their ORCID iD within the submission system before final acceptance, as unverified ORCID iDs will not appear in the published article. *Only*  the individual author can complete the verification step; PLOS staff *cannot*  verify ORCID iDs on behalf of authors.

We look forward to receiving your revised manuscript.

Kind regards,

Silvia Naitza, Ph.D

Academic Editor

PLOS One

Journal Requirements:

If the reviewer comments include a recommendation to cite specific previously published works, please review and evaluate these publications to determine whether they are relevant and should be cited. There is no requirement to cite these works unless the editor has indicated otherwise.

Please review your reference list to ensure that it is complete and correct. If you have cited papers that have been retracted, please include the rationale for doing so in the manuscript text, or remove these references and replace them with relevant current references. Any changes to the reference list should be mentioned in the rebuttal letter that accompanies your revised manuscript. If you need to cite a retracted article, indicate the article’s retracted status in the References list and also include a citation and full reference for the retraction notice.

Additional Editor Comments :

Dear Dr. Yamauchi,

Plese find attached the review with further requests to be addressed before publication of your article, suggested by one referee.

Best regards

[Note: HTML markup is below. Please do not edit.]

Reviewers' comments:

Reviewer's Responses to Questions

**Comments to the Author**

Reviewer #1: All comments have been addressed

Reviewer #2: All comments have been addressed

**********

2. Is the manuscript technically sound, and do the data support the conclusions?

Reviewer #1: Yes

Reviewer #2: Yes

**********

3. Has the statistical analysis been performed appropriately and rigorously?

Reviewer #1: Yes

Reviewer #2: Yes

**********

4. Have the authors made all data underlying the findings in their manuscript fully available?

The PLOS Data policy

Reviewer #1: Yes

Reviewer #2: Yes

**********

5. Is the manuscript presented in an intelligible fashion and written in standard English?

Reviewer #1: Yes

Reviewer #2: Yes

**********

Reviewer #1: (No Response)

Reviewer #2: This is a well-conceived, clinically relevant study addressing a real gap — how to assess hypothyroidism severity and treatment adequacy when TSH is uninformative (e.g., central hypothyroidism). The FT3/FT4 ratio is a plausible, low-cost, generalizable biomarker, and using congenital hypothyroidism (CH) as a "clean" natural experiment (defined onset, known treatment timing) is a smart study design choice. The core finding — ratio rises with severity, falls with LT4 treatment — is coherent with the deiodinase physiology discussed. However, there are several issues around internal consistency, statistical transparency, and language polish that should be addressed before publication.

**********

what does this mean?). If published, this will include your full peer review and any attached files.

If you choose “no”, your identity will remain anonymous but your review may still be made public.

**Do you want your identity to be public for this peer review?**  For information about this choice, including consent withdrawal, please see our Privacy Policy

Reviewer #1: No

Reviewer #2: **Yes:**  Murshed Ahamed Khan

**********

[NOTE: If reviewer comments were submitted as an attachment file, they will be attached to this email and accessible via the submission site. Please log into your account, locate the manuscript record, and check for the action link "View Attachments". If this link does not appear, there are no attachment files.]

To ensure your figures meet our technical requirements, please review our figure guidelines: https://journals.plos.org/plosone/s/figures

You may also use PLOS’s free figure tool, NAAS, to help you prepare publication quality figures: https://journals.plos.org/plosone/s/figures#loc-tools-for-figure-preparation.

NAAS will assess whether your figures meet our technical requirements by comparing each figure against our figure specifications.

Attachment Submitted filename: Yamauchi_Revised_Manuscript 260721.docx

Attachment Submitted filename: Reviewer Comments.docx

# Author comment: Author response to Decision Letter 2

**DOI:** 10.1371/journal.pone.0361192.r004

16 Sep 2026

We have included our response to Reviewer #2's comments in the “Response to Reviewers” file.

Attachment Submitted filename: Responce_to_Reviewer_Comments_260914.docx

# Peer review: Decision Letter 2

**DOI:** 10.1371/journal.pone.0361192.r005

**Authors:** Silvia Naitza

8 Oct 2026

Serum FT3/FT4 ratio may indicate the degree of T3 compensation in congenital hypothyroidism

PONE-D-26-09218R2

Dear Dr.a { text-decoration: none; color: #464feb;}tr th, tr td { border: 1px solid #e6e6e6;}tr th { background-color: #f5f5f5;}Yamauchi,

We’re pleased to inform you that your manuscript has been judged scientifically suitable for publication and will be formally accepted for publication once it meets all outstanding technical requirements.

Within one week, you’ll receive an e-mail detailing the required amendments. When these have been addressed, you’ll receive a formal acceptance letter and your manuscript will be scheduled for publication.

An invoice will be generated when your article is formally accepted. Please note, if your institution has a publishing partnership with PLOS and your article meets the relevant criteria, all or part of your publication costs will be covered. Please make sure your user information is up-to-date by logging into Editorial Manager at Editorial Manager® and clicking the ‘Update My Information' link at the top of the page. For questions related to billing, please contact billing support.

If your institution or institutions have a press office, please notify them about your upcoming paper to help maximize its impact. If they’ll be preparing press materials, please inform our press team as soon as possible -- no later than 48 hours after receiving the formal acceptance. Your manuscript will remain under strict press embargo until 2 pm Eastern Time on the date of publication. For more information, please contact onepress@plos.org.

Kind regards,

Silvia Naitza, Ph.D

Academic Editor

PLOS One

Additional Editor Comments (optional):

Dear Dr. Yamauchi,

Thank you for submitting the revised version of your manuscript entitled “Serum FT3/FT4 ratio may indicate the degree of T3 compensation in congenital hypothyroidism” (Manuscript ID: PONE-D-26-09218R2).

I have carefully evaluated the revised manuscript, the responses to the reviewers' comments, and the latest review. The concerns raised during the review process have been satisfactorily addressed, and the revisions have significantly strengthened the manuscript.

The study presents clinically relevant findings regarding the potential utility of the serum FT3/FT4 ratio as an indicator of T3 compensation in congenital hypothyroidism. The objectives, methodology, analyses, and interpretation of the results are clearly presented and support the conclusions drawn.

I am therefore pleased to inform you that your manuscript is accepted for publication in PLOS ONE. I would like to thank you for your careful revisions and for your constructive engagement throughout the peer-review process.

We look forward to seeing your work published and believe it will be of interest to clinicians and researchers working in pediatric endocrinology and thyroid physiology.

Sincerely,

Dr. Silvia Naitza

Academic Editor

PLOS ONE

Reviewers' comments:

Reviewer's Responses to Questions

**Comments to the Author**

Reviewer #2: (No Response)

**********

2. Is the manuscript technically sound, and do the data support the conclusions?

Reviewer #2: Yes

**********

3. Has the statistical analysis been performed appropriately and rigorously?

Reviewer #2: Yes

**********

4. Have the authors made all data underlying the findings in their manuscript fully available?

The PLOS Data policy

Reviewer #2: Yes

**********

5. Is the manuscript presented in an intelligible fashion and written in standard English?

Reviewer #2: Yes

**********

Reviewer #2: This study explored an association between CH severity and the serum FT3/FT4 ratio. It is very interesting from a clinical standpoint. Further large-scale study is needed considering the limitations of your study, validate your findings and establish definitive cutoff values of FT3/FT4 ratio to cosider as a diagnostic tool.

**********

what does this mean?). If published, this will include your full peer review and any attached files.

If you choose “no”, your identity will remain anonymous but your review may still be made public.

**Do you want your identity to be public for this peer review?**  For information about this choice, including consent withdrawal, please see our Privacy Policy

Reviewer #2: **Yes:**  Murshed Ahamed Khan

**********

Attachment Submitted filename: Reviewer Comments.docx

# Editor report: Acceptance letter

**DOI:** 10.1371/journal.pone.0361192.r006

**Authors:** Silvia Naitza

PONE-D-26-09218R2

PLOS One

Dear Dr. Yamauchi,

I'm pleased to inform you that your manuscript has been deemed suitable for publication in PLOS One. Congratulations! Your manuscript is now being handed over to our production team.

At this stage, our production department will prepare your paper for publication. This includes ensuring the following:

* All references, tables, and figures are properly cited

* All relevant supporting information is included in the manuscript submission,

* There are no issues that prevent the paper from being properly typeset

You will receive further instructions from the production team, including instructions on how to review your proof when it is ready. Please keep in mind that we are working through a large volume of accepted articles, so please give us a few days to review your paper and let you know the next and final steps.

Lastly, if your institution or institutions have a press office, please let them know about your upcoming paper now to help maximize its impact. If they'll be preparing press materials, please inform our press team within the next 48 hours. Your manuscript will remain under strict press embargo until 2 pm Eastern Time on the date of publication. For more information, please contact onepress@plos.org.

You will receive an invoice from PLOS for your publication fee after your manuscript has reached the completed accept phase. If you receive an email requesting payment before acceptance or for any other service, this may be a phishing scheme. Learn how to identify phishing emails and protect your accounts at https://explore.plos.org/phishing.

If we can help with anything else, please email us at customercare@plos.org.

Thank you for submitting your work to PLOS One and supporting open access.

Kind regards,

PLOS One Editorial Office Staff

on behalf of

Dr. Silvia Naitza

Academic Editor

PLOS One
